# The diversity and plasticity of descending motor pathways rewired after stroke and trauma in rodents

**DOI:** 10.3389/fncir.2025.1566562

**Published:** 2025-03-21

**Authors:** Takahiro Inoue, Masaki Ueno

**Affiliations:** Department of System Pathology for Neurological Disorders, Brain Research Institute, Niigata University, Niigata, Japan

**Keywords:** axon reorganization, corticospinal tract, neural circuit plasticity, red nucleus, reticular formation, spinal cord injury, spinal interneurons, stroke

## Abstract

Descending neural pathways to the spinal cord plays vital roles in motor control. They are often damaged by brain injuries such as stroke and trauma, which lead to severe motor impairments. Due to the limited capacity for regeneration of neural circuits in the adult central nervous system, currently no essential treatments are available for complete recovery. Notably, accumulating evidence shows that residual circuits of the descending pathways are dynamically reorganized after injury and contribute to motor recovery. Furthermore, recent technological advances in cell-type classification and manipulation have highlighted the structural and functional diversity of these pathways. Here, we focus on three major descending pathways, namely, the corticospinal tract from the cerebral cortex, the rubrospinal tract from the red nucleus, and the reticulospinal tract from the reticular formation, and summarize the current knowledge of their structures and functions, especially in rodent models (mice and rats). We then review and discuss the process and patterns of reorganization induced in these pathways following injury, which compensate for lost connections for recovery. Understanding the basic structural and functional properties of each descending pathway and the principles of the induction and outcome of the rewired circuits will provide therapeutic insights to enhance interactive rewiring of the multiple descending pathways for motor recovery.

## Introduction

Descending motor pathways originate from a diverse and broadly distributed neuronal population in the brain that projects axons to the spinal cord and plays crucial roles in motor control. The execution of complex movements is achieved by the sophisticated integration of these pathways to selectively activate target muscles via spinal interneurons (INs) and motor neurons (MNs) (Lemon, 2008; Arber and Costa, 2018). Importantly, this intricate motor pathway is often damaged by various forms of injury in the central nervous system (CNS), which leads to motor deficits. For instance, brain injuries such as stroke and trauma often disrupt the corticospinal pathway, resulting in severe motor impairments (Christiansen and Perez, 2018; Lin et al., 2019; Campos et al., 2023). Once the descending pathways are damaged, full regeneration of the damaged circuit is challenging; thus, no treatments currently exist for complete recovery. Nevertheless, accumulating evidence shows that residual pathways that escape injury dynamically reorganize to compensate for the injured circuit, leading to modest spontaneous recovery over time (Murphy and Corbett, 2009; Joy and Carmichael, 2021). Therefore, an effective method to promote such a rewiring process in the descending pathways may become a promising therapeutic approach for motor recovery.

Recent technological advances have enabled a comprehensive classification of the descending pathways, highlighting their anatomical and transcriptomic diversity (Wang et al., 2018; Beine et al., 2022; Wang et al., 2022; Golan et al., 2023; Winter et al., 2023). Given their diversity, reorganization is conceivably induced via multiple pathways after injury to influence the recovery process. In this review, we focus on three major descending pathways that serve as neural substrates for recovery and summarize current knowledge of their fundamental structures (i.e., origin, spinal projection, and synaptic targets) and functions. We then review and discuss the patterns of reorganization of these pathways following stroke and other traumatic injuries. Understanding the basic structural and functional properties of each pathway, including their similarities and differences, as well as the principles of the induction and functional outputs of the reorganized circuits, will provide better insights for therapeutic approaches, especially for those promoting interactive rewiring of multiple descending pathways for motor recovery.

## The diversity and major origins of descending pathways to the spinal cord

The existence of diverse descending pathways has been elucidated by retrograde tracing from the spinal cord. Nudo and Masterton (1988) investigated the origins of descending pathways in 22 mammalian species and identified 27 brain regions that were common across the species. These highly conserved structures suggest that they comprise basic neural components that cooperatively send commands for movements generally evoked in animals. In the first parts of this review, we summarize the findings of the structures and functions of the descending system, primarily focusing on those in rodents (mice and rats), which are among the most widely used mammalian models in the neuroscience field. Indeed, rodents serve as excellent models for studying neural mechanisms of movements such as reaching and locomotion, which are highly homologous to those in humans (Sacrey et al., 2009; Klein et al., 2012).

**FIGURE 1:**
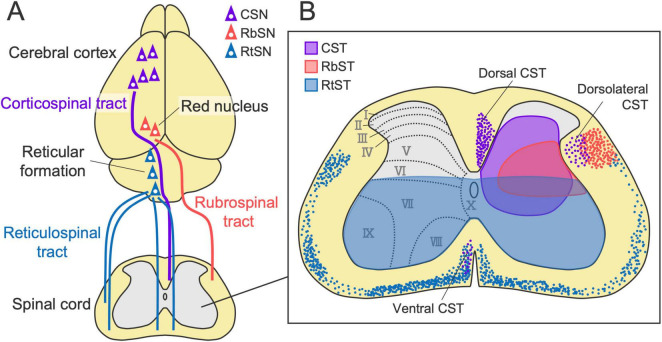
Major descending pathways to the spinal cord. **(A)** The corticospinal tract (CST), rubrospinal tract (RbST), and reticulospinal tract (RtST), originating from the cerebral cortex, red nucleus, and reticular formation. **(B)** Axon projection patterns of each pathway in the spinal cord (left-sided origins). Colored dots and shaded regions (violet, red, and blue) indicate descending axons and the typical projection area of each pathway, respectively. CST and RbST axons mainly descend in the dorsal and lateral columns on the contralateral side of their origins, respectively. RtST axons descend bilaterally in the ventral and lateral columns with a predominance of ipsilateral projections. CST, RbST, and RtST axons mainly innervate laminae III–VIII, V–VI, and VII–X, respectively. CSN, corticospinal neuron; RbSN, rubrospinal neuron; RtSN, reticulospinal neuron.

Liang et al. (2011) performed retrograde tracing by injecting conventional neuronal tracers, Fluoro-Gold and horseradish peroxidase, into the spinal cord of mice and investigated the distribution of retrogradely labeled cells throughout the entire brain. A thorough investigation revealed numerous origins of descending axons that are widely distributed across the forebrain, midbrain, and hindbrain. This finding is further supported by recent comprehensive analyses using retrograde viral tracing combined with tissue clearing and 3D imaging, which identified 69 brain regions comprising approximately 30,000 neurons that project to the spinal cord (Wang et al., 2022). These studies indicated that the primary labeled regions are the cerebral cortex (forebrain), red nucleus (midbrain), and reticular formation (hindbrain). Importantly, each region forms a major descending pathway essential for motor control: the corticospinal tract (CST), rubrospinal tract (RbST), and reticulospinal tract (RtST) (Figure 1A). We thus focused on these three representative pathways in this review, although the other pathways originating from relatively minor populations, such as those in the hypothalamus, lateral vestibular nuclei, and deep cerebellar nuclei, also play important roles in locomotion, postural balance control, and skilled movements (Low et al., 2018; Murray et al., 2018; Becker and Person, 2019; Reinoß et al., 2020; Sathyamurthy et al., 2020).

## Structures and functions of the corticospinal tract in motor control

**FIGURE 2:**
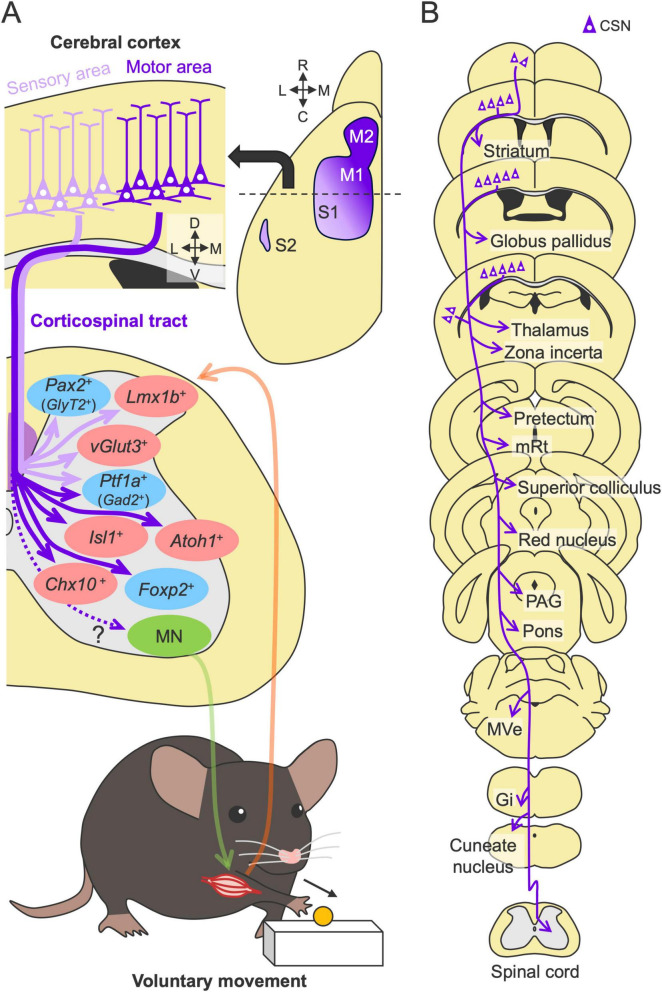
Structures and functions of the CST in motor control. **(A)** Multiple CST pathways with different cortical origins and spinal terminations. The axons from the sensory and motor cortices target dorsal (light purple arrows) and intermediate and ventral side of the spinal gray matter (violet arrows), respectively, and connect to distinct spinal interneurons (red and blue, excitatory and inhibitory neurons) for sensory and motor control [for example, Ueno et al. (2018)]. They are integrated to control efferent (green) and afferent (orange) pathways between the spinal cord and muscle. M1, primary motor cortex; M2, secondary motor cortex; S1, primary somatosensory cortex; S2, secondary somatosensory cortex; MN, motor neuron. **(B)** Subcortical collaterals of the CST. Note additional target regions beyond those shown here. mRt, mesencephalic reticular nucleus; PAG, periaqueductal gray; MVe, medial vestibular nucleus; Gi, gigantocellular reticular nucleus.

The CST is one of the most extensively studied descending pathways. The CST originates from layer V (deeper Vb) of the cerebral cortex, which forms axon bundles that project subcortically through the internal capsule, the cerebral peduncle, and the pyramid (Figures 1A, 2). The majority of the axons then cross the midline at the pyramidal decussation of the caudal medulla and project to the spinal cord predominantly contralateral to their cortical origin, mainly through the dorsal column in rodents. They then send extensive collaterals to the spinal gray matter, mainly laminae III–VIII, in rodents [(Rouiller et al., 1991; Ueno et al., 2018; Aizawa et al., 2019; Pourchet et al., 2021; Sinopoulou et al., 2022) reviewed in Welniarz et al. (2017)] (Figure 1B). A minor part of the CST axons also descends in the lateral and ventral columns. In addition, some other populations project to the ipsilateral side of their cortical origin without crossing at the pyramidal decussation or by recrossing within the spinal cord (Brosamle and Schwab, 1997; Bareyre et al., 2005; Steward et al., 2021). Most CST axon terminals contain vesicular glutamate transporter 1 (vGlut1) (Maier et al., 2008; Du Beau et al., 2012), indicating a pure excitatory pathway.

Importantly, the CST is not a simple pathway but rather includes multiple pathways comprising different cortical origins and spinal terminations. In rodents, retrograde tracing has revealed that the CST mainly arises from the primary motor (M1), primary somatosensory (S1), secondary motor (M2), and secondary somatosensory (S2) areas (Liang et al., 2011; Figure 2A). These regions are often classified based on their functional associations with specific limb movements: the rostral forelimb area (RFA), the caudal forelimb area (CFA), and the hindlimb area (Tennant et al., 2011; Steward et al., 2021). Other minor neuronal origins, such as those in the parietal, cingulate, visual, and prefrontal cortices, have also been determined (Miller, 1987; Chen et al., 2014). Like rodents, macaque monkeys have multiple cortical origins [14 regions (Darian-Smith et al., 1996)]. Recent human studies using fiber tractography have also identified more than 10 cortical origins of the CST (Seo and Jang, 2013; Chenot et al., 2019; Usuda et al., 2022), implying fundamental sensorimotor functions of these conserved multiple pathways in the CST.

Anterograde tracing has further shown that the CST of each cortical origin has distinct patterns of axon projections in the spinal gray matter. For instance, the axons from the somatosensory cortex predominantly target the dorsal horn area, whereas those from the motor areas project mainly to the intermediate and ventral areas of the spinal cord [(Kuang and Kalil, 1990; Bareyre et al., 2002; Ueno et al., 2018; Kameda et al., 2019; Steward et al., 2021) reviewed in Welniarz et al. (2017)] (Figure 2A). The axons from each cortical origin further exhibit a specific projection pattern across the spinal levels (Wang et al., 2017; Sahni et al., 2021b; Steward et al., 2021; Wang et al., 2022). Corticospinal neurons (CSNs) in the M1 and S1 extensively project from the cervical to the lumbar or sacral levels, whereas the CSNs in the RFA and S2 predominantly project to the cervical level. Lumbar-projecting CSNs are concentrated in the caudal medial part of M1 and nested within a region of cervically projecting CSNs (Kamiyama et al., 2015; Steward et al., 2021). Collectively, the anatomical studies indicate that the CST is organized by multiple CSN subpopulations that exhibit distinct projection patterns to the spinal cord.

### Corticospinal neurons have distinct synaptic targets depending on their cortical origin

The CST also comprises distinct spinal terminations among the subpopulations and species. The corticospinal axons in higher-order primates have monosynaptic connections to the spinal MNs, which are believed to function in refined motor control, such as in dexterous movements [reviewed in Lemon (2008), Alstermark and Isa (2012)]. In contrast, direct corticomotoneuronal connections are rare in other mammals, including rodents; instead, the axons typically terminate on spinal INs [reviewed in Lemon (2008), Ebbesen and Brecht (2017)]. These findings suggest that fundamental motor programs governed by the CST are encoded primarily in connections with INs in the spinal cord. Interestingly, early postnatal mice and rats initially form direct corticomotoneuronal connections, but they are eliminated in the following 2 weeks during postnatal development (Maeda et al., 2016; Gu et al., 2017). Notably, some studies have shown that a minor direct anatomical connection is also preserved in adults, as shown by conventional and recent viral tracing techniques (Liang et al., 1991; Raineteau et al., 2002; Ruigrok et al., 2008; Fageiry et al., 2024; Fait et al., 2024). This finding requires further careful anatomical and functional investigation.

Spinal INs are highly diverse and are classified into dozens of neuronal subtypes based on genetic, developmental, morphological, physiological, and neurochemical aspects [(Osseward et al., 2021; Russ et al., 2021) reviewed in Goulding (2009), Alaynick et al. (2011), Zholudeva et al. (2021)]. Given the unique multiple CST projections from different cortical origins, we and others have investigated the types of INs that receive inputs from each CST subpopulation. The analyses revealed that each projection connects to genetically distinct INs (Figure 2A). Specifically, the axons from the motor cortex predominantly connect to intermediate–ventral spinal populations such as *Chx10*^+^, *Atoh1*^+^, and *Isl1*^+^ INs that directly connect to MNs for motor outputs, whereas those in the sensory cortex connect to dorsal sensory populations such as excitatory *Vglut3*^+^ and *Lmx1b*^+^ and inhibitory *GlyT2*^+^ and *Pax2*^+^ INs (Ueno et al., 2018; Frezel et al., 2020; Fageiry et al., 2024). CSNs in the motor and sensory cortices also connect to inhibitory *Ptf1a*^+^ and *Gad2*^+^ INs that engage in presynaptic inhibition of sensory afferents (Ueno et al., 2018; Fageiry et al., 2024). Using a viral approach combining anterograde transsynaptic labeling and single-nucleus RNA sequencing (AnteroT-seq), Carmona et al. (2024) recently determined that CSNs from the RFA and CFA preferentially innervate ventral inhibitory INs (likely corresponding to *Foxp2*^+^ INs). Given these diverse types of connections, CST axons plausibly send not only motor commands to activate muscles but also multimodal information, such as for motor inhibition and gain control of sensory inputs and the spinal reflex, to regulate complex voluntary movements.

### CSN subpopulations engage in distinct sensorimotor functions during complex voluntary movements

The CST contributes to sensorimotor function, which is supported by previous studies showing that damage to the CST caused by stroke, spinal cord injury (SCI), or experimental transection called pyramidotomy leads to motor impairments [(Starkey et al., 2005; Serradj et al., 2023) reviewed in Alstermark and Isa (2012), Jones and Adkins (2015), Jones (2017)]. Interestingly, genetic ablation or optogenetic modulation of whole CSNs impairs skilled movements (e.g., forelimb reaching) but does not impair simple movements, locomotion (walking), or muscle strength, suggesting that the CST is particularly required for the accurate execution of skilled motor tasks (Wang et al., 2017; Serradj et al., 2023). Consistently, calcium imaging revealed that CSNs are typically activated during tasks (Wang et al., 2017; Serradj et al., 2023). In rodent studies, a single-pellet reaching task, which consists of 10 steps involving digit preparation, advance of the forelimb, grasping, and release, is often used to evaluate skilled forelimb movements [(Farr and Whishaw, 2002) reviewed in Nicola et al. (2022)]. How each CSN subpopulation orchestrates such complex movements is under investigation. For example, silencing *Chx10*^+^ V2a INs, the major target of the CST from the motor cortex, as well as injury in the motor cortex, impaired the advance step of the reaching task, whereas silencing *Vglut3*^+^ INs, the major target of the CSNs from the sensory cortex, as well as injury in the sensory cortex, mainly affected the releasing step (Azim et al., 2014; Ueno et al., 2018). The CSNs connected to *Chx10*^+^ V2a INs are activated just prior to and during the movement initiation, whereas sensory CSNs connected to *SST*^+^ neurons are activated thereafter (Fageiry et al., 2024). These findings suggest that the CSN subpopulations in the motor and sensory cortices have different functions in skilled movement. He’s group further identified distinct functions of the CSN subpopulations of the RFA and CFA in the execution of skilled forelimb movements (Wang et al., 2017). RFA-CSNs ablation caused deficits in grasp and pronation, whereas CFA-CSNs ablation affected aim, advance, and pronation, indicating that CFA- and RFA-CSNs play distinct roles in reaching and grasping, with shared roles in the pronation step. CST axons from the sensory cortex and those projecting to the lumbar cord have additional functions in modulating sensory information (Liu et al., 2018a; Moreno-Lopez et al., 2021) and locomotion (Karadimas et al., 2020). Taken together, these findings suggest that each CSN subpopulation regulates distinct but some overlapping aspects of voluntary movement.

### Diverse subcortical axon collaterals of corticospinal neurons

Another notable feature complicating the CST function is that the axons also send multiple collaterals to the subcortical areas along their descending route (Figure 2B). Single axon tracing revealed that descending CST axons extend intermediate collaterals to multiple subcortical regions (Kita and Kita, 2012). Recent intersectional approaches using double viral vectors enable the selective labeling and manipulation of a specific pathway, including the CST [(Kinoshita et al., 2012; Sooksawate et al., 2013; Schwarz et al., 2015) reviewed in Luo et al. (2018), Isa (2022)]. For instance, injections of a retrograde virus encoding Cre recombinase into the spinal cord and another recombinase-dependent reporter virus into the cerebral cortex can specifically label the CSNs, which can further label axons and collaterals extending to diverse subcortical regions, such as the striatum, globus pallidus, thalamus, zona incerta, tectum, mesencephalic reticular nucleus (mRt), superior colliculus, red nucleus (RN), periaqueductal gray (PAG), pons, medial vestibular nucleus (MVe), gigantocellular reticular nucleus (Gi), and cuneate nucleus (Wang et al., 2017; Frezel et al., 2020; Brain Initiative Cell Census Network (BICCN), 2021; Nelson et al., 2021; Lu et al., 2022; Sinopoulou et al., 2022; Carmona et al., 2024; Figure 2B). Interestingly, many of the above regions are known to have subsequent descending projections to the spinal cord (Liang et al., 2011; Wang et al., 2022), suggesting that the CST may also transmit commands to the parallel descending pathways to the spinal cord through the axon collaterals. Importantly, despite the presence of such broadly distributed collaterals, approximately 90% of synaptic termini appear to converge in the spinal cord (Wang et al., 2017).

The functional significance of this broadcasting network, especially in sensorimotor control, still remains to be determined [reviewed in Arber and Costa (2018)]. A recent study investigated the connections of the CST collaterals to the striatum, which receives the most abundant collaterals among the subcortical areas in rodents (Nelson et al., 2021). The study identified two CSN subpopulations that innervate distinct types of striatal neurons, as well as the spinal INs, whereas these populations exhibited similar activity patterns during the forelimb motor task (Nelson et al., 2021). These findings imply that (i) each CSN module conveys similar information to downstream motor executive circuits through this broadcasting system and that (ii) collateral-receiving postsynaptic circuits play roles in forming more specific aspects of movements. Outside the striatum, subcortical neuronal subtypes that receive collateral inputs and their subsequent connections and roles in motor control remain largely unknown. Although additional studies are needed to understand the functional properties of these cortico-subcortical pathways to the spinal cord, this unique parallel descending system would be beneficial for compensating for lost motor functions when one pathway is injured, which is discussed in later sections.

## Structures and functions of the rubrospinal tract in motor control

**FIGURE 3:**
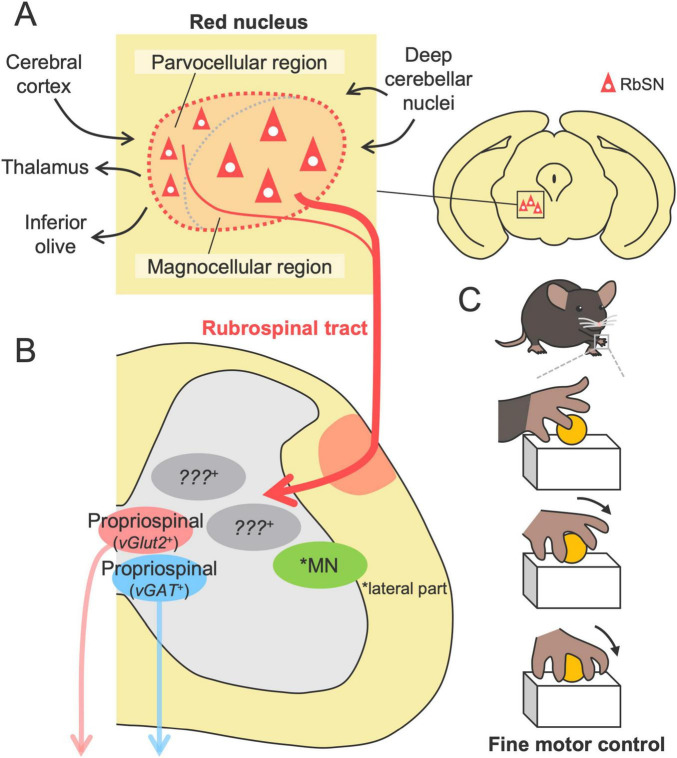
Structures and functions of the RbST in motor control. **(A)** The red nucleus comprising the parvocellular and magnocellular regions, the origins of the RbST (upper, left). Afferents from the cerebral cortex and deep cerebellar nuclei and efferents to the inferior olive, thalamus, and spinal cord. **(B)** RbST axons connect to excitatory and inhibitory spinal interneurons, while their genetic profile remains unknown. **(C)** The RbST is suggested to contribute to fine motor control, such as distal dexterous motion in the reaching task.

The red nucleus (RN), a distinct neuronal cluster located in the midbrain, has been investigated over the past century; however, research on the RN has waned, despite its importance in the motor system [reviewed in Massion (1967, 1988), Basile et al. (2021), Olivares-Moreno et al. (2021)]. The RN is cytoarchitectonically divided into two subregions: the rostral parvocellular region, consisting of small- to medium-sized neurons, and the caudal magnocellular region, consisting of large, sparse neurons, which form somewhat unclear boundaries in rodents [(Reid et al., 1975; Strominger et al., 1987; Liang et al., 2012) reviewed in Basile et al. (2021)] (Figure 3). The absolute populations of magnocellular and parvocellular neurons are approximately equal in number in rodents (Aghoghovwia and Oorschot, 2016). The RN further includes a third population, interneurons, which are widely distributed in the RN and have much smaller size than the other neurons (Strominger et al., 1987).

The rubrospinal tract (RbST), the descending pathway from the RN, has many properties that are similar to those of the CST. The primary origin is the magnocellular region, with partial contributions from the parvocellular region (Murray and Gurule, 1979; Huisman et al., 1981; Shieh et al., 1983; Strominger et al., 1987; Liang et al., 2011, 2012; Figure 3). RbST axons cross the midline within the midbrain (the ventral tegmental decussation), then descend in the lateral column of the spinal cord, and project to the lateral part of the intermediate zone (mainly laminae V-VI) contralateral to their origin (Brown, 1974b; Yasui et al., 2001; Liang et al., 2012; Figure 1). Small ipsilateral innervations were also identified for ipsilaterally descending or recrossing axons in the spinal cord (Shieh et al., 1983; Antal et al., 1992; Küchler et al., 2002; Liang et al., 2012). Although the RbST projects widely along the spinal level (Strominger et al., 1987; Antal et al., 1992), cervical-projecting rubrospinal neurons (RbSNs) topographically exist in the dorsomedial part of the RN, whereas lumbar-projecting neurons in the ventral region and thoracic-projecting neurons in the intermediate part have been identified (Murray and Gurule, 1979; Strominger et al., 1987; Wang et al., 2022). Some RbSNs co-innervate the contralateral side of the reticular formation and the cerebellum with collaterals (Huisman et al., 1983; Yasui et al., 2001). Most RbST axon terminals contain vesicular glutamate transporter 2 (vGlut2) (Du Beau et al., 2012), indicating an excitatory pathway. The diverse origins, contralateral-dominant innervation, topographical organization, and multiregional collaterals are typical features shared with the CST.

The majority of the RbST axons are suggested to terminate onto spinal INs of both excitatory and inhibitory subtypes, as demonstrated by RN lesion experiments, anterograde tracing, electron microscopy, and immunohistochemical staining (Brown, 1974b; Antal et al., 1992; Figure 3A). Recent monosynaptic rabies tracing demonstrated that RbSNs connect to both excitatory *vGlut2*^+^ and inhibitory *vGAT*^+^ long descending propriospinal INs (Ruder et al., 2016). Anatomical and electrophysiological studies have further shown that minor RbST axons also directly connect to MNs in rodents (Raineteau et al., 2001; Küchler et al., 2002). The connections preferentially target dendrites rather than the soma of the MNs that predominantly control intermediate and distal muscles (forearm and digits) but not the proximal muscles. A single RbST axon innervates multiple MNs (Küchler et al., 2002). Notably, such direct connections have also been demonstrated by monosynaptic rabies tracing in mice (Esposito et al., 2014). However, a comprehensive understanding of the connections between RbSNs and spinal neurons remains limited.

The current knowledge of RbST function is mainly based on lesion studies. Damage to the RbST had little effect on general locomotion and the success rate of the reaching task but significantly impaired the substeps of the reaching task, including aiming, pronation and supination (Whishaw et al., 1990; Whishaw et al., 1992; Whishaw et al., 1998; Muir and Whishaw, 2000; Kanagal and Muir, 2008; Morris et al., 2011; Morris et al., 2015; Rizzi et al., 2019; Figure 3C). This finding was further confirmed by the chemogenetic silencing of RN neurons (Rizzi et al., 2019), suggesting that the RbST contributes to fine motor control rather than gross control. In particular, “arpeggio movement,” characterized by sequential movements of digits during the pronation step of the reaching task (Whishaw and Gorny, 1994), appears to be a unique function mediated by the RbST. This movement is impaired in RN lesions or RbST transection but not in CST transection or partial lesions of the parvocellular region [(Whishaw et al., 1998; Kanagal and Muir, 2008; Morris et al., 2011; Morris et al., 2015) reviewed in Morris and Whishaw (2016)]. These findings suggest that the control of distal dexterous motion relies on the magnocellular-derived RbST. This hypothesis is further supported by anatomical and physiological RbST connections to the distal muscle MNs (Küchler et al., 2002) and RN firing evoked immediately before the end of the reaching movement, likely corresponding to the pronation step (Jarratt and Hyland, 1999). However, it should be noted that a chemical lesion of the RN and surgical transection of the RbST may cause impairments beyond the function of the RbST. For instance, RN ablation can also disrupt efferent pathways to the inferior olive and thalamus, as well as the afferents such as the corticorubral tract from the cerebral cortex and those from the deep cerebellar nuclei [(Brown, 1974a; Swenson and Castro, 1983; Akintunde and Buxton, 1992; Lopez-Virgen et al., 2022) reviewed in Basile et al. (2021)] (Figure 3A).

In addition to the traditional anatomical segregations, genetic characterization of RbSNs has begun to be revealed. Using retrograde labeling, *in situ* hybridization, and immunohistochemistry, Liang et al. (2012) identified *vGlut2*^+^ and *C1ql2*^+^ neurons in regions of retrogradely labeled RbSNs and suggested that SMI-32 may be a potential marker for caudal RbSNs. Recently, retrograde labeling from the lumbar cord and single-nucleus RNA sequencing (snRNA-seq) by Blackmore’s group identified marker genes of RbSNs, such as *Rreb1*, *Emx2*, and *Ttc6* (Beine et al., 2022). A more recent study by He’s group performing retrograde labeling from a more extensive region of the spinal cord and snRNA-seq has further revealed genetic signatures that distinguish the RbSN subpopulation (Winter et al., 2023). They found that *Pvalb*, *Kcng4*, and *Spp1* are expressed in most RbSNs, whereas their expression levels differ among the subpopulations, which are related to their cell size and electrophysiological properties. *Spp1*^–^ RbSNs are present primarily in the rostral area, likely corresponding to the parvocellular region, whereas *Spp1*^+^ RbSNs are likely present in the magnocellular region (Winter et al., 2023). The genetic classification may enable the development of genetic and viral tools to label and manipulate specific RbSN subpopulations, which will lead to a deeper understanding of the RbST structure and functions.

## Structures and functions of the reticulospinal tract in motor control

**FIGURE 4:**
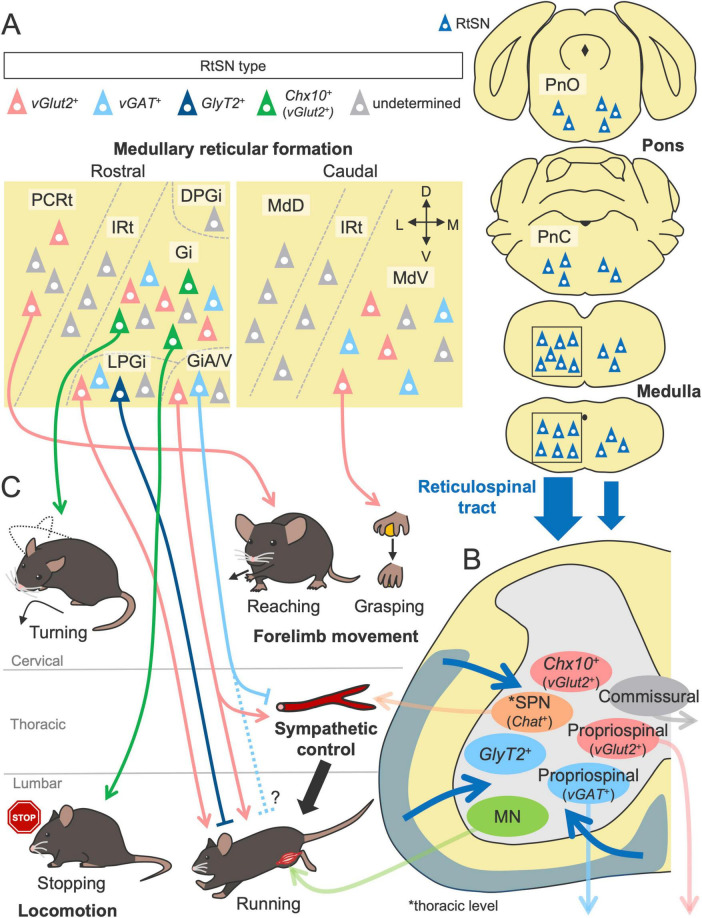
Structures and functions of the RtST in motor control. **(A)** Multiple nuclei in the reticular formation as the origins of the RtST. The gigantocellular reticular nucleus (Gi) in the medullary reticular formation is the primary origin of the RtST, while the pontine reticular formation (oral and caudal pontine reticular nucleus; PnO and PnC) and the midbrain reticular formation are the moderate and minor origins, respectively. **(B)** Spinal INs [commissural INs, long descending propriospinal neurons, and sympathetic preganglionic neurons (SPNs)] and motor neurons (MNs) are connected by the RtST. **(C)** RtSN subpopulations regulate distinct aspects of voluntary movement and locomotion (left, bottom). Inhibitory and excitatory RtSNs exert opposite effects. The alpha/ventral gigantocellular reticular nucleus (GiA/V) co-regulate motor and sympathetic functions. Please see the text for details. DPGi, dorsal paragigantocellular nucleus; LPGi, lateral paragigantocellular nucleus; IRt, intermediate reticular nucleus; PCRt, parvicellular reticular nucleus; MdV, ventral medullary reticular nucleus; MdD, dorsal medullary reticular nucleus.

The reticulospinal tract (RtST) is one of the most complex descending pathways because its origin, the reticular formation (RF), comprises multiple neural nuclei distributed across the brainstem, including the midbrain, pons and medulla (Figure 4). The RF exhibits an ambiguous structure, which lacks clear cytoarchitectonic boundaries; thus, the taxonomy and nomenclature of each nucleus are often inconsistent across literatures and species (Brownstone and Chopek, 2018; Perreault and Giorgi, 2019). Many RF nuclei have variable amounts of descending projections to the spinal cord, but the majority of RtST, especially the gigantocellular reticular nucleus (Gi, also known as the GRN), primarily arises from the medullary RF (Reed et al., 2008; Liang et al., 2011; Wang et al., 2022; Figure 4A). The pontine RF also moderately contributes to the origin of the RtST, whereas the projections of the midbrain RF appear to be relatively low [(Huma et al., 2014) reviewed in Perreault and Giorgi (2019)].

The complexity of the RF is further increased by the presence of various subtypes of neurons defined by neurotransmitters, such as glutamatergic, GABA/glycinergic, monoaminergic, and cholinergic neurons [reviewed in Ruder and Arber (2019), Arber and Costa (2022)]. These neurons are genetically characterized by the expression of unique transcription factors during development (Gray, 2013). Thus, unlike the pure excitatory systems in the CST and RbST, the RtST contains various types of neurotransmitters and modulators (VanderHorst and Ulfhake, 2006; Du Beau et al., 2012; Hossaini et al., 2012; Mitchell et al., 2016; Capelli et al., 2017; Zhang et al., 2024). This finding highlights the functional diversity and multimodal roles of the RtST.

In contrast to the predominant contralateral innervation of the CST and RbST, the RtST predominantly projects to the ipsilateral side of the spinal cord with weak lateralization, as shown by retrograde tracing from the spinal cord (Liang et al., 2011; Sivertsen et al., 2016; Valencia Garcia et al., 2018; Pourchet et al., 2021; Figure 1). Anterograde tracing further showed that medulla-derived RtST axons project bilaterally to the ventral spinal cord (mainly laminae VII-X) but with a predominance of ipsilateral projections across all the spinal levels (Jones and Yang, 1985; Martin et al., 1985; Liang et al., 2015; Capelli et al., 2017; Cregg et al., 2020). Ipsilateral dominance is also observed in pons-derived RtST axons (Jones and Yang, 1985; Liang et al., 2016).

Like CSNs and RbSNs, reticulospinal neurons (RtSNs) of different RF origins have specific projection patterns to the spinal cord. For example, RtSNs in the medial part of the pontine RF project to the ipsilateral spinal cord, whereas a small population in the lateral part projects contralaterally, as observed in the tracing study of neonatal mice (Sivertsen et al., 2016). Physiological studies have further demonstrated that electrical stimulation of the medullary RF activates specific commissural INs and distinct motor columns in the spinal cord, depending on the stimulation site [(Szokol et al., 2008, 2011) reviewed in Perreault and Glover (2013)]. Interestingly, recent genetic and viral experiments have shown that RtSNs, even within a particular region, exhibit distinct projection patterns to the spinal gray matter, depending on their neuronal subtype (Capelli et al., 2017; Zhang et al., 2024). These findings suggest that RtSNs are organized with functionally diverse subpopulations that are intermingled in each RF region.

Although a comprehensive investigation has not yet been conducted, RtSNs are reported to connect to various types of spinal neurons (Figure 4B). MNs are one of the targets, which have been validated primarily by anatomical experiments (Zagon and Bacon, 1991; Hermann et al., 2003; Capelli et al., 2017; Xie et al., 2023). Using transsynaptic viral tracing in mice, Arber’s group further determined that premotor RtSNs, which are monosynaptically connected to MNs, are broadly distributed across RF nuclei, such as the Gi, magnocellular reticular nucleus (Mc), medullary RF ventral part (MdV), parvicellular reticular nucleus (PCRt), and pontine reticular nucleus (Pn) (Esposito et al., 2014; Figure 4A). Notably, each premotor RtSN population selectively connects to functionally distinct MNs. For instance, RtSNs of the MdV primarily connect to forelimb MNs rather than hindlimb MNs, with a marked preference for biceps MNs over triceps MNs. Although the premotor RtSNs in the MdV are excitatory *vGlut2*^+^ neurons (Esposito et al., 2014), their subsequent work revealed that an inhibitory *GlyT2*^+^ subpopulation in the lateral paragigantocellular nucleus (LPGi) preferentially targets the MNs more than the *vGlut2*^+^ RtSNs do (Capelli et al., 2017). Indeed, another group has described numerous inhibitory termini on the proximal dendrites of MNs using electron microscopy (Holstege, 1987, 1991; Holstege and Bongers, 1991).

Commissural INs and long descending propriospinal neurons have also been identified as postsynaptic targets of RtSNs in anatomical and physiological studies [(Szokol et al., 2011; Mitchell et al., 2016; Ruder et al., 2016) reviewed in Perreault and Glover (2013)] (Figure 4B). Importantly, these neurons are known to form monosynaptic connections with MNs (Ni et al., 2014; Goetz et al., 2015) and contribute to left–right and forelimb–hindlimb coordination in locomotion [reviewed in Laliberte et al. (2019)]. A comparative study revealed that commissural INs and long descending propriospinal neurons receive minimal contacts from the CSNs but many inputs from the RtSNs, with approximately 80% excitatory and 20% inhibitory connections (Mitchell et al., 2016). As other synaptic targets, excitatory *vGlut2*^+^ (Esposito et al., 2014), inhibitory *vGAT*^+^ (Esposito et al., 2014) and *vGlyT2*^+^ INs (Bouvier et al., 2015), and sympathetic preganglionic neurons (Aicher et al., 1995; Zhang et al., 2024) have been identified. Thus, RtSNs form excitatory and inhibitory connections with MNs and spinal INs, which could be a substrate for generating and controlling diverse types of movement. Future studies are expected to further characterize the genetic and functional features of target spinal neurons.

Based on the variable connections to the spinal neurons, the RtST would mediate multimodal functions in motor control (Figure 4C). Like the CST and RbST, the RtST also contributes to skilled forelimb movement. This finding is supported by evidence that ablation of the MdV, one of the origins of the RtST, impairs the performance of the grasping phase in the forelimb reaching task (Esposito et al., 2014). Their subsequent work demonstrated that the PCRt, referred to as the LatRM (lateral rostral medulla), is also important for forelimb movement, as its firing rate increased specifically during the forelimb task (Ruder et al., 2021). Chemogenetic silencing of excitatory PCRt neurons impaired performance in the reaching phase of the task, and optogenetic stimulation of spinally projecting excitatory PCRt neurons evoked a forelimb reaching movement but not a digit movement (Ruder et al., 2021). These findings indicate that each RtSN subpopulation regulates distinct aspects of voluntary movement.

Notably, in contrast to the minimal influence of the CST and RbST on locomotion (Wang et al., 2017; Rizzi et al., 2019), the RtST plays pivotal roles in locomotor control (Figure 4C). Kiehn’s group found that optogenetic activation of excitatory *Chx10*^+^ RtSNs in the Gi suppressed locomotor rhythm generation and activity via inhibitory spinal INs [(Bouvier et al., 2015; Cregg et al., 2020) reviewed in Leiras et al. (2022)]. In addition, a recent study identified projection-specific subpopulations of *Chx10*^+^ Gi-RtSNs: cervical-projecting neurons influence head orientation and the locomotor trajectory by directly innervating MNs, whereas lumbar-projecting neurons reduce the locomotor speed without turning orientation (Usseglio et al., 2020). A most recent study revealed that the *Chx10*^+^ Gi neurons is driven by inputs from excitatory Pn neurons within the RF (especially in the oral part: PnO) (Cregg et al., 2024). Other excitatory LPGi neurons initiate forward-directed locomotion and sustain high-speed locomotion, but these effects are abolished when inhibitory LPGi neurons are co-activated by unconditional optogenetic stimulation (Capelli et al., 2017). The high-speed locomotion is also modulated by excitatory RtSNs in the alpha/ventral part of the Gi (GiA/V) (Zhang et al., 2024). Interestingly, GiA/V-RtSNs innervate not only the lumbar premotor area but also thoracic sympathetic preganglionic neurons, enabling co-regulation of somatomotor and sympathetic functions that supports the high-speed locomotion (Zhang et al., 2024). Like the LPGi, the intermingled inhibitory RtSNs in the GiA/V play an opposite role to the action of excitatory RtSNs (Zhang et al., 2024). Taken together, these findings indicate that RtSN subpopulations are highly organized to regulate distinct aspects of locomotor movement, enabling fine behavioral adjustments through the selective activation of each subpopulation (Figure 4).

For details of the role of the reticulospinal system in motor control, please refer to other excellent reviews, which include findings from other species [reviewed in Brownstone and Chopek (2018), Ruder and Arber (2019), Arber and Costa (2022)].

## Rewiring of the descending pathways and recovery after stroke and trauma

Given the importance of descending pathways in motor control, as described above, their damage undoubtedly results in diverse types of motor impairment. Regenerating the original pathway is highly challenging due to the limited capacity for axon growth and neurogenesis in the adult CNS [reviewed in Lie et al. (2004), Liu et al. (2011), He and Jin (2016), Mahar and Cavalli (2018)]. However, although full recovery is rare, a modest level of spontaneous recovery is often observed over time. This recovery has been demonstrated to be achieved by the reorganization of spared neural circuits functionally linked to the damaged circuits, thereby compensating for the lost functions [reviewed in Murphy and Corbett (2009), Jones and Adkins (2015), Campos et al. (2023)].

**FIGURE 5:**
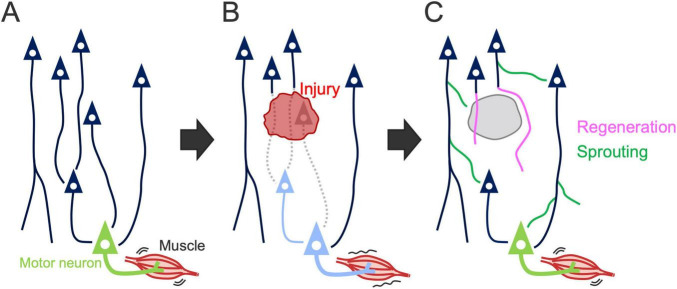
Axon rewiring to recover motor function after injury. **(A)** Illustration showing the descending motor circuits. **(B)** Descending pathways are often disrupted by the injury, resulting in motor dysfunction. Dashed lines, damaged axons; light blue neurons, denervated motor and premotor neurons. **(C)** Spared neurons rewire their axons to compensate for the lost circuit, leading to motor recovery. Axon growth in the rewiring process is categorized into “regeneration” arising from the tips of injured axons (pink lines) and “sprouting” originating from the middle of injured or uninjured axons (green lines) (Tuszynski and Steward, 2012; Geoffroy and Zheng, 2014).

The process of reorganization involves various plastic events of residual neurons, including structural and functional remodeling of axons, dendrites, and synapses, which underlie changes in gene expression and neural activity [reviewed in Murphy and Corbett (2009), Joy and Carmichael (2021), Campos et al. (2023), Jones et al. (2024)]. Axonal regrowth is an important process that supports reorganization and functional recovery after stroke and SCI [(Weidner et al., 2001; Ueno et al., 2012) reviewed in Benowitz and Carmichael (2010), Fink and Cafferty (2016), Hollis (2016), Carmichael et al. (2017)]. Axonal regrowth can be categorized into two distinct processes: “regeneration” arising from the tips of injured axons and “sprouting” originating from the middle of injured or uninjured axons (Tuszynski and Steward, 2012; Geoffroy and Zheng, 2014; Figure 5). Here, we summarize the patterns of axon rewiring that occur in the descending pathways, especially when the CST is damaged. Specifically, we address three forms of CST injury: cortical, subcortical, and spinal cord injury (SCI), in which cortical injury directly damages the CSNs, whereas subcortical injury and SCI damage the descending axonal tract but remain CSN cell bodies intact.

### Rewiring of the descending pathways following cortical injury

**FIGURE 6:**
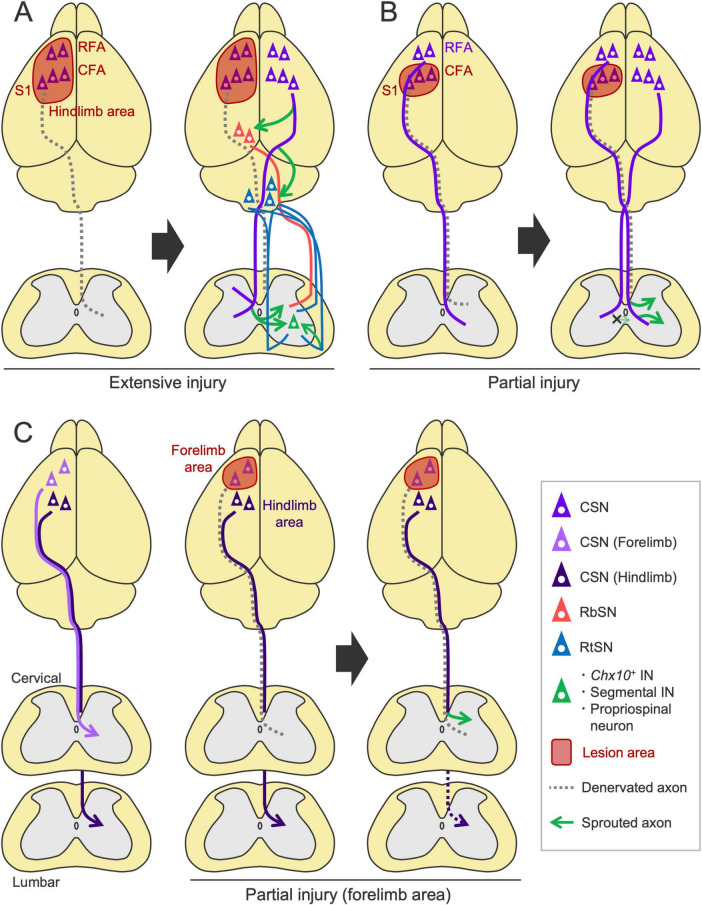
Rewiring patterns of the descending pathways following cortical injury. **(A)** After a large injury to the sensorimotor cortex, contralesional CST axons sprout into the denervated side of the spinal cord (Lee et al., 2004; Ueno et al., 2012; Bachmann et al., 2014; Kaiser et al., 2019; Sato et al., 2021) and connect to *Chx10*^+^ INs that would include segmental INs and propriospinal neurons (Ueno et al., 2012; Sato et al., 2025). Corticorubral, corticoreticular, RbST and RtST projections are also enhanced in the denervated areas (Chen et al., 2002; Lee et al., 2004; Bachmann et al., 2014; Liu et al., 2015). **(B)** In a small injury limited to the caudal forelimb area (CFA) and S1, CST axons from the ipsilesional rostral forelimb area (RFA) sprout on the denervated side, while the axons from the contralesional CFA do not (Okabe et al., 2016; Okabe et al., 2017a; Sato et al., 2021). **(C)** Following stroke in the forelimb area, CSNs of the ipsilesional hindlimb area enhance cervical projections (Starkey et al., 2012b).

Damage to CSNs is primarily observed in cortical injuries, such as stroke and trauma. In this case, rewiring is mediated by axon sprouting from spared neurons. We and others have shown that, after unilateral injury to the sensorimotor cortex, intact CST axons originating from the contralesional cortex newly sprout into the denervated side of the spinal cord and contribute to motor recovery (Lee et al., 2004; Liu et al., 2008; Lapash Daniels et al., 2009; Ueno et al., 2012; Kaiser et al., 2019; Sato et al., 2021; Figure 6A). These sprouting axons connect with specific spinal INs, such as segmental INs and propriospinal neurons (Ueno et al., 2012), which play critical roles in motor control [reviewed in Isa et al. (2007)]. Recent study has further identified *Chx10*^+^ V2a INs as the target neurons (Sato et al., 2025). Interception or chemogenetic silencing of intact CST fibers as well as the target *Chx10*^+^ INs impaired the recovered motor functions, indicating the importance of the rewired pathway in recovery (Ueno et al., 2012; Wahl et al., 2014; Sato et al., 2025). These models show 20–30% spontaneous recovery in reaching task and the interception of the residual network mostly disrupt the recovered portion, indicating the functional importance of the rewired circuit (Ueno et al., 2012; Sato et al., 2025). This finding is further supported by electrophysiological responses and retrograde tracing, which showed an increase in ipsilaterally projecting CSNs (Ueno et al., 2012; Bachmann et al., 2014; Lindau et al., 2014).

Importantly, the population of CSNs recruited in the rewiring process varies depending on the lesion size and location. In a large stroke involving the RFA, CFA and S1 in which the CST originates, the contralesional CST axons sprout as described above, but this process does not occur in a small stroke where the lesion is limited to the CFA and S1 (Sato et al., 2021; Figure 6B). Instead, the axons from the ipsilesional RFA sprout on the denervated side (Sato et al., 2021). Another study reported that ipsilesional S2-CSNs increase spinal projections following stroke (Bachmann et al., 2014). These findings reveal the basic principles that generate the patterns of CST rewiring: ipsilesional CSNs are predominantly recruited for rewiring, and contralesional CSNs are involved when the ipsilesional CSNs are mostly eliminated by the injury (Figures 6A, B). In support of these findings, rodent evidence has demonstrated that inactivation of the contralesional cortex by lidocaine has minimal effects on rehabilitation-induced recovery in a small stroke but significantly disrupts it in a large stroke (Biernaskie et al., 2005). Other studies have documented the significance of spared ipsilesional areas in motor recovery after a relatively small stroke or injury, as shown by electrophysiological assays or secondary lesion experiments of the spared areas (Nishibe et al., 2010; Nishibe et al., 2015; Okabe et al., 2016; Okabe et al., 2017a; Abe et al., 2018). These findings indicate that the contribution of the ipsi- and contralesional cortex to recovery depends on the lesion size, as discussed in human patients (Cramer et al., 2011; Dodd et al., 2017). Additional factors such as severity of the injury may also determine the patterns of the rewiring process, which should be further investigated.

Generally, functionally homologous CSNs compensate for the damaged circuit; however, some redundancies are reported among the different cortical areas and pathways. For instance, following stroke in the sensorimotor cortex of the forelimb area, CSNs in the hindlimb area compensate for the lost cervical projections of the forelimb CSNs (Starkey et al., 2012b; Figure 6C). Interestingly, these hindlimb CSNs withdraw their original projections from the lumbar cord and instead innervate the cervical cord (Starkey et al., 2012b). Although recent DNA microarray and snRNA-seq studies identified *Epha4*, *Epha6*, *Epha7*, *Efna5*, *Crim1*, and *Klhl14* as candidate genes underlying the cervical- and lumbar-projecting properties (Sahni et al., 2021a; Winter et al., 2023), how these molecules are involved in switching their spinal projection targets after injury remains unclear. Importantly, the CST subpopulation derived from the motor and sensory cortices does not mutually innervate the dorsal and ventral spinal areas, respectively, when the sensory or motor cortex is injured (Sato et al., 2021). This finding suggests that functionally different subpopulations of the CST cannot be rewired to compensate for each other and implies that some strong molecular cues restrict areal projections within the dorsal and ventral spinal cord (Gu et al., 2019).

Reorganization is observed not only in the spared CST but also in other spared pathways. For instance, corticorubral and corticoreticular projections from the contralesional cortex are enhanced after stroke (Chen et al., 2002; Lee et al., 2004; Bachmann et al., 2014; Liu et al., 2015; Figure 6A). Other subcortical projections, such as the corticostriatal axons, are also enhanced (Napieralski et al., 1996; Carmichael and Chesselet, 2002). In other cases, however, spontaneous sprouting does not occur in these pathways (Papadopoulos et al., 2002; Lee et al., 2013; Choi et al., 2018; Zhang et al., 2018). This discrepancy may depend on the experimental conditions, such as the lesion size, type, and location. Regarding the RbST, retrograde tracing has shown that the number of labeled RbSNs does not change after a cortical stroke (Bachmann et al., 2014; Okabe et al., 2018). Although whether RbST axons sprout after stroke has not yet been examined, a mouse model with a congenital absence of the CST has been reported to exhibit an increase in the spinal projections of RbST axons (Han et al., 2015). Retrograde tracing studies further indicated that RtSNs in the Gi increase their projections to the denervated side of the spinal cord following stroke (Bachmann et al., 2014; Okabe et al., 2018; Figure 6A). Since both the RbST and RtST contain some midline-crossing axons in the spinal cord under normal conditions (Antal et al., 1992; Liang et al., 2016), rewiring to switch the innervation side may also occur in the RbST and RtST, similar to the CST. No studies have systematically examined the rewiring patterns of CST, RbST and RtST axons across different stroke conditions.

### Rewiring of the descending pathways following subcortical injury

**FIGURE 7:**
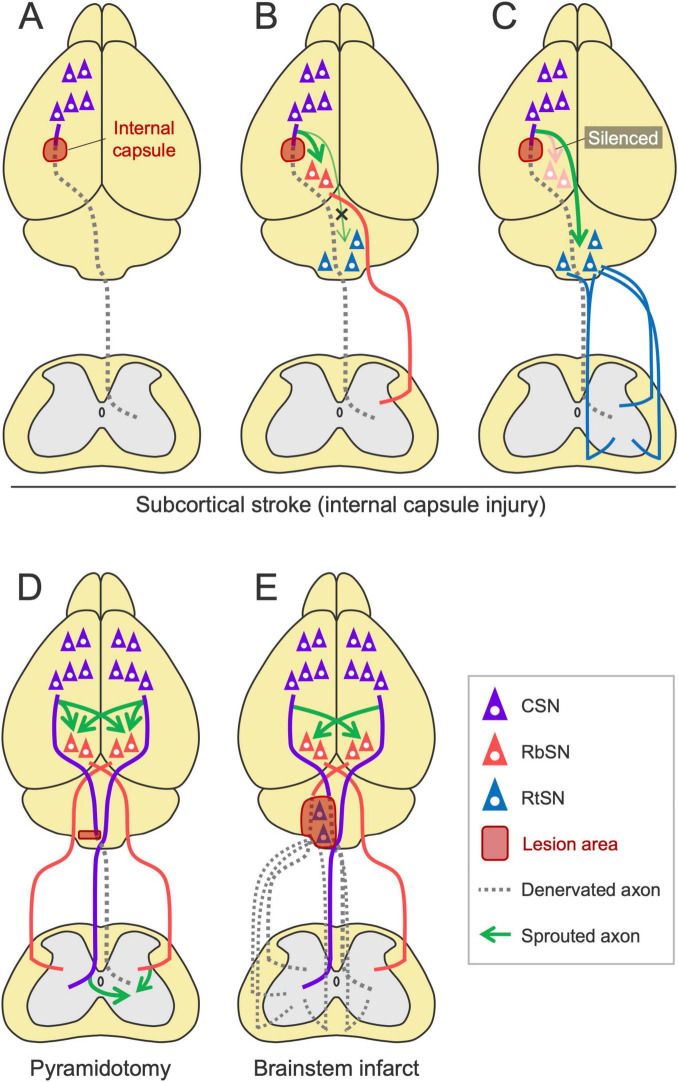
Rewiring patterns of the descending pathways following subcortical injury. **(A)** Subcortical stroke, such as internal capsule hemorrhage, damages the CST. **(B)** The ipsilesional corticorubral projection is enhanced by rehabilitation (Ishida et al., 2016; Ishida et al., 2019). **(C)** The corticoreticular projection is enhanced when the corticorubral pathway is silenced (Ishida et al., 2019). **(D)** In pyramidotomy, RbST axons sprout in the spinal cord (Siegel et al., 2015). Rehabilitation enhances CST collateral projections to the red nucleus (Mosberger et al., 2018). Corticorubral, RbST, and CST crossing projections are also enhanced when anti-Nogo-A antibody is treated (Thallmair et al., 1998; Z’Graggen et al., 1998; Raineteau et al., 2001; Raineteau et al., 2002). **(E)** A brainstem infarct increases bilateral corticorubral projections to the contralateral side (Minnerup et al., 2018). Note that this model would damage not only the RtST but also the CST and RbST.

Subcortical infarcts and intracerebral hemorrhages often damage the CST pathway. In rodents, injury to the internal capsule, a frequent site of hemorrhagic stroke that the CST passes, is widely used and results in chronic motor deficits (Liu et al., 2018b; Inoue et al., 2021; Figure 7A). The rewiring has been observed in these models especially when the plastic process is enhanced by rehabilitation or inhibition of axon growth inhibitors such as Nogo-A. Using a rat model and viral tools, Ishida et al. (2016, 2019) demonstrated a causal link between the rewiring of the descending pathways and functional recovery induced by rehabilitation. They showed that the intensive use of the impaired forelimb promotes motor recovery and increases the ipsilesional corticorubral projections to both the parvocellular and magnocellular regions of the RN but not to the Gi and MdV of the RF (Ishida et al., 2016; Ishida et al., 2019; Figure 7B). Selective silencing of the corticorubral pathway abolished rehabilitation-induced motor recovery, characterized by kinematic deficits such as elbow extension and wrist rotation in the forelimb reaching task, but preserved digit movements and coordinated locomotor control (Ishida et al., 2016). In this case, 50%–60% of recovery was induced in the reaching test and the silencing mostly abolished the recovered portion (Ishida et al., 2016; Ishida et al., 2019). In support of these results, a study using pyramidotomy have shown that rehabilitation enhances the sprouting of injured CST collateral projections in the RN and that the selective silencing of the RbSNs impairs rehabilitation-induced recovery (Mosberger et al., 2018; Figure 7D). In addition, RbST axons spontaneously sprout in the spinal cord after pyramidomy (Siegel et al., 2015). The loss of CST inputs in the spinal cord by pyramidotomy is compensated by sprouting RbST and contralateral CST axons following anti-Nogo-A antibody (IN-1) treatment [(Thallmair et al., 1998; Raineteau et al., 2001; Raineteau et al., 2002), reviewed in Raineteau and Schwab (2001)]. The IN-1 treatment also increases corticorubral projections to the contralesional RN (Thallmair et al., 1998; Z’Graggen et al., 1998). In neonatal injuries, these types of rewiring spontaneously occur (Z’Graggen et al., 2000; Liu et al., 2010), although corticorubral projections are not increased in another case (Wen et al., 2018). Electrical stimulation of the contralesional cortex also enhances corticorubral projections to the bilateral RN (Carmel et al., 2013). Taken together, when the CST pathway is damaged, the RN plays a vital role in mediating recovery. CSNs likely switch their transmission to the RbST to restore the lost spinal connections and their functions.

Notably, when the corticorubral pathway is experimentally silenced during rehabilitation, corticoreticular inputs instead increase and contribute to functional recovery (Ishida et al., 2019; Figure 7C). Consistently, in both CST and RbST injuries in rats, rehabilitation combined with chondroitinase-ABC administration increases residual RtST axon sprouting into the spinal gray matter devoid of CST and RbST inputs (Garcia-Alias et al., 2015). These findings indicate the compensatory role of the RtST and the hierarchical priority of these pathways in reorganization. How injured CSNs or spared cortical neurons determine the alternative pathway and how the RN and RF attract these novel projections remain unknown.

The rewiring process when the RbST or RtST is injured is not sufficiently examined. Notably, a rodent model of brainstem stroke has been established recently, showing increased crossing fibers of the bilateral corticorubral tracts at the RN level (Minnerup et al., 2018; Figure 7E). It should be noted, however, that this model damages not only the RtST but also the CST and RbST due to the anatomical location (Minnerup et al., 2018). Another study showed that RbST ablation by RN lesions did not induce sprouting of residual CST axons (Jeffery and Fitzgerald, 2001). Considering that midbrain stroke is rare in clinical settings (Kumral et al., 2002), the RN and RbST that remain intact could be key substrates for rewiring and motor recovery after stroke.

### Rewiring of the descending pathways following spinal cord injury

**FIGURE 8:**
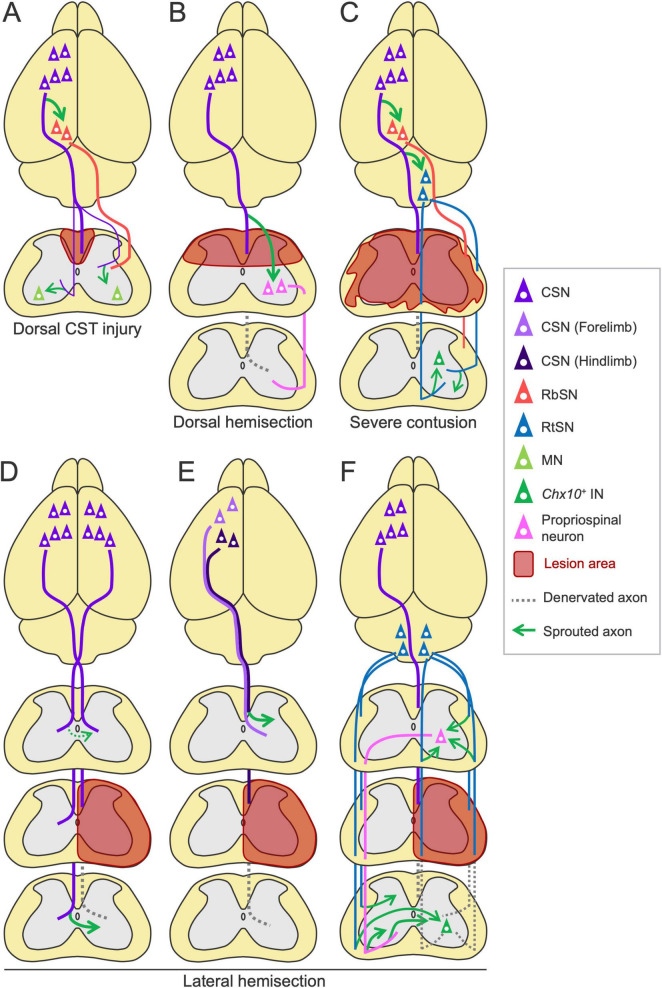
Rewiring patterns of the descending pathways following spinal cord injury. **(A)** In the dorsal CST injury, the minor dorsolateral and ventral CST axons sprout and connect to the motor neurons (MNs) (Weidner et al., 2001; Bareyre et al., 2005; Hilton et al., 2016). Rehabilitation enhances the corticorubral projection (Sheikh et al., 2024). **(B)** In the dorsal hemisection, residual CST axons sprout and connect to descending propriospinal neurons, creating a detour route to the caudal region (Bareyre et al., 2004; van den Brand et al., 2012; Bradley et al., 2019; Van Steenbergen et al., 2023). **(C)** In severe contusion injury, rehabilitation increases corticoreticular and corticorubral projections and preserved RtST axon collaterals in the spinal cord, connecting to *Chx10*^+^ INs (Asboth et al., 2018; Kathe et al., 2022). **(D–F)** Rewiring patterns in the lateral hemisection. **(D)** Spared CST axons from the ipsilesional cortex sprout to the denervated side in the caudal area to the lesion, but do limitedly in the rostral area (Ghosh et al., 2009; van den Brand et al., 2012; Collyer et al., 2014; Friedli et al., 2015). **(E)** Injured lumbar-projecting CST axons from the hindlimb area (dark violet) sprout in the cervical cord, rostral to the thoracic lesion (Fouad et al., 2001; Ghosh et al., 2010). **(F)** Spared contralesional RtST axons sprout to the denervated side in the caudal area to the lesion (Ballermann and Fouad, 2006; Zorner et al., 2014). In the rostral area, injured ipsilesional RtST axons sprout and connect to double-midline crossing propriospinal neurons to bypass the lesion, which also increase their collateral sprouting (Filli et al., 2014).

Spinal cord injury is another type of injury that frequently damages the CST pathway. Descending pathways are also reorganized in this case, in which the patterns of rewiring vary depending on the type and spinal level of the injury [reviewed in Anderson et al. (2022)] (Figure 8). For instance, in a dorsal hemisection model, spared CST axons at the rostral level sprout to connect with descending propriospinal neurons, creating detour/relay pathways that bridge the injury site [(Bareyre et al., 2004; van den Brand et al., 2012; Bradley et al., 2019; Van Steenbergen et al., 2023) reviewed in Anderson et al. (2022)] (Figure 8B). In a lateral hemisection model, spared contralesional CST axons cross the midline to the denervated side caudal to the injury site, but they limitedly cross in the rostral area [(Ghosh et al., 2009; van den Brand et al., 2012; Collyer et al., 2014; Friedli et al., 2015) reviewed in Anderson et al. (2022)] (Figure 8D). The minor population, dorsolateral and ventral CST axons also contribute to the rewiring and recovery by increasing connections to the MNs when the major dorsal CST is damaged (Weidner et al., 2001; Bareyre et al., 2005; Hilton et al., 2016; Figure 8A). Corticorubral projections are also enhanced by rehabilitation (Sheikh et al., 2024; Figure 8A). In another case, after injury at the thoracic level, axotomized lumbar-projecting CST axons from the hindlimb area of the cerebral cortex change their projections to the cervical cord and are incorporated into the sensorimotor circuits controlling the forelimb (Fouad et al., 2001; Ghosh et al., 2010; Figure 8E). Similar interactive rewiring between the forelimb and hindlimb CSNs has been observed in stroke (Starkey et al., 2012b; Figure 6C), implying the existence of a shared mechanism beyond the types of injury. In SCI models, more than 60% of recovery is observed and silencing or transection of rewired circuits mostly decreases the recovered portion, indicating functional importance of the rewired circuits (Weidner et al., 2001; Bareyre et al., 2004; Bradley et al., 2019).

Courtine’s group recently demonstrated the importance of the RtST after SCI (Figures 8C, F). First, they showed that spinal cord contusion injury mostly destroys the CST and RbST but relatively preserves the RtST, owing to its widely dispersed projections in the spinal white matter (Asboth et al., 2018). They further found that glutamatergic RtSNs in the ventral Gi relay cortical commands to the spinal cord and mediate hindlimb muscle activity and that rehabilitation increases both the sprouting of corticoreticular and Gi-RtSN projections (van den Brand et al., 2012; Asboth et al., 2018). Additionally, rehabilitation likely enhances corticorubral projections in this model (Asboth et al., 2018; Figure 8C). In their later work, they reported that Gi-derived RtST axons connect to *Vsx2*^+^ (*Chx10*^+^) INs in the spinal cord (Kathe et al., 2022; Squair et al., 2023; Figures 8C, F). Chemogenetic silencing of each pathway or specific *Vsx2*^+^ INs disrupts the recovery of walking induced by rehabilitation (Kathe et al., 2022; Squair et al., 2023). These findings indicate the importance of rewired corticoreticular and reticulospinal circuits in recovery. Their most recent study showed that Gi-RtSNs also relay inputs from the lateral hypothalamus (Cho et al., 2024). Other groups further showed that after a lateral hemisection injury, spared contralesional RtST axons grow to cross the midline to the denervated region caudal to the lesion (Ballermann and Fouad, 2006; Zorner et al., 2014), while ipsilesional RtST axons sprout and form excitatory connections to double-midline crossing propriospinal neurons in the rostral side to bypass the injury site (Filli et al., 2014; Figure 8F). The double-midline crossing propriospinal neurons also increase their sprouting and contribute to the recovery (Courtine et al., 2008; Filli et al., 2014). These findings indicate the compensatory role of the RtST in SCI.

## Perspectives to promote rewiring for recovery

In this review, we summarized the findings of three descending motor pathways and their interactive rewiring, which occurs after injuries in rodent models. Each pathway has the following features to contribute to recovery:

(1) Heterogeneity—multiple distinct pathways (and neuron types) intermingled within each tract;
(2) Redundancy—the structure and function partially overlap with those of the other pathways;
(3) Flexibility—they are capable of switching to alternative routes when disrupted.

Thus, promoting the growth and rewiring of residual descending axons is the first promising approach to achieve recovery. Since most of the reports indicate that spontaneous or intervention-induced rewiring is effective but not yet sufficient for complete recovery to pre-injury level in cortical injury (Ueno et al., 2012; Bachmann et al., 2014; Lindau et al., 2014), subcortical injury (Siegel et al., 2015; Ishida et al., 2016; Mosberger et al., 2018), and SCI models (Nakamura et al., 2021; Kathe et al., 2022), we further discuss future research directions as below.

### Overcoming intrinsic and extrinsic factors inhibiting axon growth

Axons have a limited ability to regrow in the adult CNS due to the inhibitory environments, such as myelin-related proteins and chondroitin sulfate proteoglycans (CSPGs), as well as the limited intrinsic capacity of adult neurons to grow axons [reviewed in Yiu and He (2006)]. Indeed, CST sprouting is dramatically decreased when the brain is injured in adults compared with injuries in the postnatal stage (Omoto et al., 2010). Many experimental trials have succeeded in promoting the growth of descending axons in adults; for example, targeting extrinsic factors by knocking out myelin-related proteins (Lee et al., 2004; Lee et al., 2010; Omoto et al., 2010; Wahl et al., 2014), deleting axon growth inhibitors such as semaphorins and RGMa (Hata et al., 2006; Omoto et al., 2011; Ueno et al., 2020), digesting CSPG (Garcia-Alias et al., 2009; Starkey et al., 2012a), and others [reviewed in Silver et al. (2014)]; and targeting intrinsic factors such as deleting PTEN, which enhances mTOR signaling (Liu et al., 2010), administering inosine (Zai et al., 2009), and overexpressing Sox11 (Wang et al., 2015). Combinatorial targeting, such as by *Pten* and *Rho* double knockouts, was also effective (Nakamura et al., 2021). A recent phase 2b clinical trial in SCI patients shows that anti-Nogo-A antibody treatment improves motor recovery in patients with incomplete injury (Weidner et al., 2025), which is supported by another clinical trial using a blocker of Nogo receptor 1 ligands (Maynard et al., 2023). Anti RGMa antibody treatment also enhances motor recovery coincided with plastic changes in the descending pathways, which is currently tested in clinical trials (Nakagawa et al., 2019; Jacobson et al., 2021). These studies suggest that treatments designed to promote axon growth may also be effective in humans, especially in cases where residual axons survive.

Importantly, for better recovery, growing axons need to reconstruct functional connections with appropriate target neurons, beyond just aberrantly promoting axon growth (Wahl et al., 2014; Anderson et al., 2018; Squair et al., 2023). For example, chemoattraction to guide the transected axons of *Vsx2*^+^ descending propriospinal neurons to their natural target *Vsx2*^+^ and *Chat*^+^ neurons led to a substantial recovery of walking after complete SCI in mice, whereas simply regenerating the axons across the lesion had no effect (Squair et al., 2023). Maladapted circuits formed by brain-derived neurotrophic factor (BDNF) transduction or compensatory innervation of sensory afferents rather induce spastic changes and worsen motor outcomes [(Lu et al., 2012; Tan et al., 2012) reviewed in Brown and Weaver (2012)].

### Understanding circuit connections to re-establish for recovery

To achieve sufficient recovery, we must gain a deeper understanding of the functional connections that each descending pathway forms and how to navigate those connections. As discussed above, intersectional approaches and transsynaptic viral tools, such as monosynaptic rabies virus (Stepien et al., 2010), adeno-associated virus (serotype 1) (Zingg et al., 2017; Zingg et al., 2020), and omics approaches (Winter et al., 2023), will be useful for elucidating the connections of diverse subpopulations of descending pathways and target neurons. Compared with those of the CST and RtST, our knowledge of the RbST appears to be limited since the analyses are still based mainly on traditional methods. The type of spinal INs that is targeted by sprouted axons and mediate recovery after SCI (Kathe et al., 2022; Squair et al., 2023) and stroke (Sato et al., 2025) have been identified, but the entire connections are not fully understood. In addition, how to re-establish those specific connections will be the next step to overcome. The treatment of synaptic organizers that promote synaptic connections may be one way to promote functional connections (Suzuki et al., 2020). Notably, CST axons from the motor cortex have the ability to selectively reinnervate appropriate spinal targets, such as *Chx10*^+^ INs, without additional exogenous guidance after SCI and stroke (Kumamaru et al., 2019; Sato et al., 2025), suggesting that some molecular cues connect appropriate sets of neurons. Integrating the effects of axon growth and synaptic connections on multiple descending pathways is an additional step to overcome.

### Identifying the critical molecules for rewiring

A compelling future direction would be to investigate the molecular mechanisms that promote the rewiring process. Several studies have attempted to identify the molecular factors that induce CST rewiring after cortical stroke or pyramidotomy using a DNA microarray analysis, but the critical molecules had not been determined (Bareyre et al., 2002; Maier et al., 2008; Zai et al., 2009). We and others identified that target-derived BDNF is involved in the reorganization of CST axons (Bareyre et al., 2002; Vavrek et al., 2006; Ueno et al., 2012; Sato et al., 2025) and corticorubral axons following neonatal brain injury (Chang et al., 2022). Other candidates, such as transforming growth factor β1 (TGF-β1), ciliary neurotrophic factor (CNTF), and lipid phosphate phosphatase-related protein type 1 (LPPR1), have also been reported to contribute to CST axon sprouting after stroke and pyramidotomy (Jin et al., 2015; Fink et al., 2017; Kaiser et al., 2019). We have recently applied cell-type specific mRNA expression analyses using RiboTag and identified that Scg2 is upregulated by injury-induced ATP signals in astrocytes and rehabilitation-induced neural activity in target *Chx10*^+^ V2a neurons in the denervated cervical cord, and drives the growth of residual CST axons after stroke (Sato et al., 2025). Cellular and molecular mechanisms that initiate axon growth and re-establish connections should be further explored to understand if the above mechanisms generally occur in denervated areas after diverse types of CNS injury. Given the distinct neuronal types of the CSN, RbSN, and RtSN (Winter et al., 2023), pathway-specific molecular programs, as well as shared growth factors that widely act across the pathways [reviewed in Weishaupt et al. (2012)], might be involved in inducing the rewiring.

### Maximizing the effect with rehabilitation

Rehabilitation is one of the most practical approaches to facilitate the reorganization of the descending pathways and recovery [reviewed in Wahl and Schwab (2014), Okabe et al. (2017b), Okabe and Miyamoto (2018)]. In terms of the molecular mechanisms, exercise induces BDNF and Scg2 expression in the brain and spinal cord and could support the induction of rewiring and recovery after stroke (Gomez-Pinilla et al., 2001; Inoue et al., 2022; Inoue et al., 2023; Sato et al., 2025). Scg2 expression is spontaneously induced after stroke over time and further enhanced by rehabilitation (Sato et al., 2025), suggesting that rehabilitation-induced rewiring builds upon spontaneous rewiring. However, the underlying mechanisms critical for promoting rewiring and establishing connections are not fully understood.

Neural activity would be involved in the rewiring process due to its enhancement after physical exercise (Chen et al., 2019). Indeed, CST sprouting is promoted by various forms of neuronal activation, such as electrical, optogenetic, and chemogenetic stimulation [(Wahl et al., 2017; Boato et al., 2023; Yang and Martin, 2023; Yang et al., 2024) reviewed in Carmel and Martin (2014)]. Consistently, neuronal activity activates intrinsic signaling pathways for axon growth, such as the mTOR pathway (Chen et al., 2019; Zareen et al., 2024). In contrast, inactivation of cortical or spinal neurons decreases CST sprouting and connections to target neurons (Lee et al., 2011; Bradley et al., 2019). Furthermore, other studies suggest that coordinated and synchronized activity support the formation of functional connections (Carmichael and Chesselet, 2002; Van Steenbergen et al., 2023), which are based on the neuronal principle of forming functional connections during development (Matsumoto et al., 2024). In the future, elucidating how the activities of the descending pathways are regulated and synchronized with those of the target neurons will be imperative. In this context, understanding how the descending pathways function as a part of the large CNS network, including the intracortical, subcortical and spinal circuits, is also necessary.

The effects of rehabilitation on reorganization and recovery vary depending on the type of training (Girgis et al., 2007; Fawcett, 2009; Maier et al., 2009; Nakagawa et al., 2013; Okabe et al., 2018). This finding implies the need to develop rehabilitative programs optimized for the target descending pathways and circuits. In particular, the type, intensity, time, and duration of rehabilitation that promote reorganization and recovery should be examined to maximize the effects of treatment in different stroke or injury conditions. Another promising strategy is to combine rehabilitation with electrical stimulation, pharmacotherapy, or genetic interventions, which could amplify the effects of rewiring and recovery (Wahl et al., 2014; Hollis et al., 2016; Wahl et al., 2017; Abe et al., 2018; Asboth et al., 2018; Inoue et al., 2020; Tanaka et al., 2020; Inoue et al., 2022; Yang et al., 2024). However, how these interventions can be combined with rehabilitation should be further investigated. For example, simultaneous treatment with an anti-Nogo-A antibody and rehabilitation resulted in aberrant growth of residual CST axons but did not induce recovery of performance in the forelimb motor task after stroke due to excessive growth and aberrant termination patterns of rewired CST axons (Wahl et al., 2014). In contrast, rehabilitation following a certain period of the antibody treatment dramatically enhanced the recovery, presumably by establishing functional connections. Considering the growth inhibitory role of Nogo-A, its antibody treatment would facilitate rewiring through a distinct process parallel to the spontaneous and rehabilitation-induced rewiring.

### Bridging a gap between rodent findings and clinical applications

Monkey studies also show reorganization of the descending pathways (Friedli et al., 2015; Morecraft et al., 2016; Darling et al., 2018; Isa et al., 2019), supporting the occurrence of plastic changes in higher primates as well. In humans, clinical imaging studies in stroke patients have successfully found microstructural changes in spared regions, including the red nucleus and reticulospinal tract, and these changes correlated with motor recovery or impairment (Ruber et al., 2012; Takenobu et al., 2014; Karbasforoushan et al., 2019). Although detecting fine changes, such as axon sprouting, remains challenging in humans, these findings suggest that circuit rewiring observed in rodents may also occur in CNS injury patients and lead to functional changes.

Although the descending pathways are conserved across species, differences in their structures and rewiring patterns should be considered. In rodents, CST axons descend through the dorsoventral funiculus of the spinal cord, whereas in humans, they descend through the dorsolateral funiculus [reviewed in Welniarz et al. (2017)]. Rodents exhibit extensive CST collaterals to multiple subcortical regions, whereas primate collaterals are restricted more predominantly to the spinal cord (Sinopoulou et al., 2022). Corticomotoneuronal connections are typical features of higher primates, including humans, whereas disynaptic connections are predominant in rodents [reviewed in Isa et al. (2007), Lemon (2008)]. The cortical size and regions comprising the CST are definitely larger in humans than in rodents. These structural differences may lead to differences in the neuronal components spared following injury, thereby contributing to the discrepancy in rewiring patterns among species (Friedli et al., 2015). Indeed, lesions in the cortex show more complicated patterns of reorganization of descending pathways in a monkey model (Morecraft et al., 2016; Darling et al., 2018; Isa et al., 2019). Rodent models are useful for exploring basic mechanisms, but further practical mechanisms and approaches for patients should also be explored in higher primates. Recognizing such a gap and the basic neuronal principles shared across species will advance the translational applications of laboratory findings into clinical practice.

## References

[B1] Abe H. Jitsuki S. Nakajima W. Murata Y. Jitsuki-Takahashi A. Katsuno Y. (2018). CRMP2-binding compound, edonerpic maleate, accelerates motor function recovery from brain damage. *Science* 360 50–57. 10.1126/science.aao2300 29622647

[B2] Aghoghovwia B. E. Oorschot D. E. (2016). Absolute number of parvicellular and magnocellular neurons in the red nucleus of the rat midbrain: A stereological study. *J. Anat.* 229 406–415. 10.1111/joa.12495 27257130 PMC4974543

[B3] Aicher S. A. Reis D. J. Nicolae R. Milner T. A. (1995). Monosynaptic projections from the medullary gigantocellular reticular formation to sympathetic preganglionic neurons in the thoracic spinal cord. *J. Comp. Neurol.* 363 563–580. 10.1002/cne.903630405 8847418

[B4] Aizawa S. Okada T. Keino-Masu K. Doan T. H. Koganezawa T. Akiyama M. (2019). Abnormal pyramidal decussation and bilateral projection of the corticospinal tract axons in mice lacking the heparan sulfate endosulfatases. Sulf1 and Sulf2. *Front. Mol. Neurosci.* 12:333. 10.3389/fnmol.2019.00333 32038163 PMC6985096

[B5] Akintunde A. Buxton D. F. (1992). Origins and collateralization of corticospinal, corticopontine, corticorubral and corticostriatal tracts: A multiple retrograde fluorescent tracing study. *Brain Res.* 586 208–218. 10.1016/0006-8993(92)91629-s 1381650

[B6] Alaynick W. A. Jessell T. M. Pfaff S. L. (2011). SnapShot: Spinal cord development. *Cell* 146 178–178.e171. 10.1016/j.cell.2011.06.038 21729788 PMC3158655

[B7] Alstermark B. Isa T. (2012). Circuits for skilled reaching and grasping. *Annu. Rev. Neurosci.* 35 559–578. 10.1146/annurev-neuro-062111-150527 22524789

[B8] Anderson M. A. O’Shea T. M. Burda J. E. Ao Y. Barlatey S. L. Bernstein A. M. (2018). Required growth facilitators propel axon regeneration across complete spinal cord injury. *Nature* 561 396–400. 10.1038/s41586-018-0467-6 30158698 PMC6151128

[B9] Anderson M. A. Squair J. W. Gautier M. Hutson T. H. Kathe C. Barraud Q. (2022). Natural and targeted circuit reorganization after spinal cord injury. *Nat. Neurosci.* 25 1584–1596. 10.1038/s41593-022-01196-1 36396975

[B10] Antal M. Sholomenko G. N. Moschovakis A. K. Storm-Mathisen J. Heizmann C. W. Hunziker W. (1992). The termination pattern and postsynaptic targets of rubrospinal fibers in the rat spinal cord: A light and electron microscopic study. *J. Comp. Neurol.* 325 22–37. 10.1002/cne.903250103 1484116

[B11] Arber S. Costa R. M. (2018). Connecting neuronal circuits for movement. *Science* 360 1403–1404. 10.1126/science.aat5994 29954969

[B12] Arber S. Costa R. M. (2022). Networking brainstem and basal ganglia circuits for movement. *Nat. Rev. Neurosci.* 23 342–360. 10.1038/s41583-022-00581-w 35422525

[B13] Asboth L. Friedli L. Beauparlant J. Martinez-Gonzalez C. Anil S. Rey E. (2018). Cortico-reticulo-spinal circuit reorganization enables functional recovery after severe spinal cord contusion. *Nat. Neurosci.* 21 576–588. 10.1038/s41593-018-0093-5 29556028

[B14] Azim E. Jiang J. Alstermark B. Jessell T. M. (2014). Skilled reaching relies on a V2a propriospinal internal copy circuit. *Nature* 508 357–363. 10.1038/nature13021 24487617 PMC4230338

[B15] Bachmann L. C. Lindau N. T. Felder P. Schwab M. E. (2014). Sprouting of brainstem-spinal tracts in response to unilateral motor cortex stroke in mice. *J. Neurosci.* 34 3378–3389. 10.1523/JNEUROSCI.4384-13.2014 24573294 PMC6795311

[B16] Ballermann M. Fouad K. (2006). Spontaneous locomotor recovery in spinal cord injured rats is accompanied by anatomical plasticity of reticulospinal fibers. *Eur. J. Neurosci.* 23 1988–1996. 10.1111/j.1460-9568.2006.04726.x 16630047

[B17] Bareyre F. M. Haudenschild B. Schwab M. E. (2002). Long-lasting sprouting and gene expression changes induced by the monoclonal antibody IN-1 in the adult spinal cord. *J. Neurosci.* 22 7097–7110. 10.1523/JNEUROSCI.22-16-07097.2002 12177206 PMC6757902

[B18] Bareyre F. M. Kerschensteiner M. Misgeld T. Sanes J. R. (2005). Transgenic labeling of the corticospinal tract for monitoring axonal responses to spinal cord injury. *Nat. Med.* 11 1355–1360. 10.1038/nm1331 16286922

[B19] Bareyre F. M. Kerschensteiner M. Raineteau O. Mettenleiter T. C. Weinmann O. Schwab M. E. (2004). The injured spinal cord spontaneously forms a new intraspinal circuit in adult rats. *Nat. Neurosci.* 7 269–277. 10.1038/nn1195 14966523

[B20] Basile G. A. Quartu M. Bertino S. Serra M. P. Boi M. Bramanti A. (2021). Red nucleus structure and function: From anatomy to clinical neurosciences. *Brain Struct. Funct.* 226 69–91. 10.1007/s00429-020-02171-x 33180142 PMC7817566

[B21] Becker M. I. Person A. L. (2019). Cerebellar control of reach kinematics for endpoint precision. *Neuron* 103 335–348.e335. 10.1016/j.neuron.2019.05.007 31174960 PMC6790131

[B22] Beine Z. Wang Z. Tsoulfas P. Blackmore M. G. (2022). Single nuclei analyses reveal transcriptional profiles and marker genes for diverse supraspinal populations. *J. Neurosci.* 42 8780–8794. 10.1523/JNEUROSCI.1197-22.2022 36202615 PMC9698772

[B23] Benowitz L. I. Carmichael S. T. (2010). Promoting axonal rewiring to improve outcome after stroke. *Neurobiol. Dis.* 37 259–266. 10.1016/j.nbd.2009.11.009 19931616 PMC2818530

[B24] Biernaskie J. Szymanska A. Windle V. Corbett D. (2005). Bi-hemispheric contribution to functional motor recovery of the affected forelimb following focal ischemic brain injury in rats. *Eur. J. Neurosci.* 21 989–999. 10.1111/j.1460-9568.2005.03899.x 15787705

[B25] Boato F. Guan X. Zhu Y. Ryu Y. Voutounou M. Rynne C. (2023). Activation of MAP2K signaling by genetic engineering or HF-rTMS promotes corticospinal axon sprouting and functional regeneration. *Sci. Transl. Med.* 15:eabq6885. 10.1126/scitranslmed.abq6885 36599003

[B26] Bouvier J. Caggiano V. Leiras R. Caldeira V. Bellardita C. Balueva K. (2015). Descending command neurons in the brainstem that halt locomotion. *Cell* 163 1191–1203. 10.1016/j.cell.2015.10.074 26590422 PMC4899047

[B27] Bradley P. M. Denecke C. K. Aljovic A. Schmalz A. Kerschensteiner M. Bareyre F. M. (2019). Corticospinal circuit remodeling after central nervous system injury is dependent on neuronal activity. *J. Exp. Med.* 216 2503–2514. 10.1084/jem.20181406 31391209 PMC6829605

[B28] Brain Initiative Cell Census Network (BICCN). (2021). A multimodal cell census and atlas of the mammalian primary motor cortex. *Nature* 598 86–102. 10.1038/s41586-021-03950-0 34616075 PMC8494634

[B29] Brosamle C. Schwab M. E. (1997). Cells of origin, course, and termination patterns of the ventral, uncrossed component of the mature rat corticospinal tract. *J. Comp. Neurol.* 386 293–303. 9295153 10.1002/(sici)1096-9861(19970922)386:2<293::aid-cne9>3.0.co;2-x

[B30] Brown A. Weaver L. C. (2012). The dark side of neuroplasticity. *Exp. Neurol.* 235 133–141. 10.1016/j.expneurol.2011.11.004 22116043 PMC4851547

[B31] Brown L. T. (1974a). Corticorubral projections in the rat. *J. Comp. Neurol.* 154 149–167. 10.1002/cne.901540204 4826092

[B32] Brown L. T. (1974b). Rubrospinal projections in the rat. *J. Comp. Neurol.* 154 169–187. 10.1002/cne.901540205 4826093

[B33] Brownstone R. M. Chopek J. W. (2018). Reticulospinal systems for tuning motor commands. *Front. Neural Circuits* 12:30. 10.3389/fncir.2018.00030 29720934 PMC5915564

[B34] Campos B. Choi H. DeMarco A. T. Seydell-Greenwald A. Hussain S. J. Joy M. T. (2023). Rethinking remapping: Circuit mechanisms of recovery after stroke. *J. Neurosci.* 43 7489–7500. 10.1523/JNEUROSCI.1425-23.2023 37940595 PMC10634578

[B35] Capelli P. Pivetta C. Soledad Esposito M. Arber S. (2017). Locomotor speed control circuits in the caudal brainstem. *Nature* 551 373–377. 10.1038/nature24064 29059682

[B36] Carmel J. B. Martin J. H. (2014). Motor cortex electrical stimulation augments sprouting of the corticospinal tract and promotes recovery of motor function. *Front. Integr. Neurosci.* 8:51. 10.3389/fnint.2014.00051 24994971 PMC4061747

[B37] Carmel J. B. Kimura H. Berrol L. J. Martin J. H. (2013). Motor cortex electrical stimulation promotes axon outgrowth to brain stem and spinal targets that control the forelimb impaired by unilateral corticospinal injury. *Eur. J. Neurosci.* 37 1090–1102. 10.1111/ejn.12119 23360401 PMC3618589

[B38] Carmichael S. T. Chesselet M. F. (2002). Synchronous neuronal activity is a signal for axonal sprouting after cortical lesions in the adult. *J. Neurosci.* 22 6062–6070. 10.1523/JNEUROSCI.22-14-06062.2002 12122067 PMC6757933

[B39] Carmichael S. T. Kathirvelu B. Schweppe C. A. Nie E. H. (2017). Molecular, cellular and functional events in axonal sprouting after stroke. *Exp. Neurol.* 287 384–394. 10.1016/j.expneurol.2016.02.007 26874223 PMC4980303

[B40] Carmona L. M. Thomas E. D. Smith K. Tasic B. Costa R. M. Nelson A. (2024). Topographical and cell type-specific connectivity of rostral and caudal forelimb corticospinal neuron populations. *Cell Rep.* 43:113993. 10.1016/j.celrep.2024.113993 38551963 PMC11100358

[B41] Chang L. Masada M. Kojima M. Yamamoto N. (2022). Involvement of denervated midbrain-derived factors in the formation of ectopic cortico-mesencephalic projection after hemispherectomy. *J. Neurosci.* 42 749–761. 10.1523/JNEUROSCI.1371-21.2021 34887319 PMC8808734

[B42] Chen K. Zheng Y. Wei J. A. Ouyang H. Huang X. Zhang F. (2019). Exercise training improves motor skill learning via selective activation of mTOR. *Sci. Adv.* 5:eaaw1888. 10.1126/sciadv.aaw1888 31281888 PMC6609215

[B43] Chen P. Goldberg D. E. Kolb B. Lanser M. Benowitz L. I. (2002). Inosine induces axonal rewiring and improves behavioral outcome after stroke. *Proc. Natl. Acad. Sci. U S A.* 99 9031–9036. 10.1073/pnas.132076299 12084941 PMC124418

[B44] Chen T. Koga K. Descalzi G. Qiu S. Wang J. Zhang L. S. (2014). Postsynaptic potentiation of corticospinal projecting neurons in the anterior cingulate cortex after nerve injury. *Mol. Pain* 10:33. 10.1186/1744-8069-10-33 24890933 PMC4060852

[B45] Chenot Q. Tzourio-Mazoyer N. Rheault F. Descoteaux M. Crivello F. Zago L. (2019). A population-based atlas of the human pyramidal tract in 410 healthy participants. *Brain Struct. Funct.* 224 599–612. 10.1007/s00429-018-1798-7 30460551

[B46] Cho N. Squair J. W. Aureli V. James N. D. Bole-Feysot L. Dewany I. (2024). Hypothalamic deep brain stimulation augments walking after spinal cord injury. *Nat. Med.* 30 3676–3686. 10.1038/s41591-024-03306-x 39623087

[B47] Choi I. A. Lee C. S. Kim H. Y. Choi D. H. Lee J. (2018). Effect of inhibition of DNA methylation combined with task-specific training on chronic stroke recovery. *Int. J. Mol. Sci.* 19:2019. 10.3390/ijms19072019 29997355 PMC6073594

[B48] Christiansen L. Perez M. A. (2018). Targeted-plasticity in the corticospinal tract after human spinal cord injury. *Neurotherapeutics* 15 618–627. 10.1007/s13311-018-0639-y 29946981 PMC6095776

[B49] Collyer E. Catenaccio A. Lemaitre D. Diaz P. Valenzuela V. Bronfman F. (2014). Sprouting of axonal collaterals after spinal cord injury is prevented by delayed axonal degeneration. *Exp. Neurol.* 261 451–461. 10.1016/j.expneurol.2014.07.014 25079366

[B50] Courtine G. Song B. Roy R. R. Zhong H. Herrmann J. E. Ao Y. (2008). Recovery of supraspinal control of stepping via indirect propriospinal relay connections after spinal cord injury. *Nat. Med.* 14 69–74. 10.1038/nm1682 18157143 PMC2916740

[B51] Cramer S. C. Sur M. Dobkin B. H. O’Brien C. Sanger T. D. Trojanowski J. Q. (2011). Harnessing neuroplasticity for clinical applications. *Brain* 134 1591–1609. 10.1093/brain/awr039 21482550 PMC3102236

[B52] Cregg J. M. Leiras R. Montalant A. Wanken P. Wickersham I. R. Kiehn O. (2020). Brainstem neurons that command mammalian locomotor asymmetries. *Nat. Neurosci.* 23 730–740. 10.1038/s41593-020-0633-7 32393896 PMC7610510

[B53] Cregg J. M. Sidhu S. K. Leiras R. Kiehn O. (2024). Basal ganglia-spinal cord pathway that commands locomotor gait asymmetries in mice. *Nat. Neurosci.* 27 716–727. 10.1038/s41593-024-01569-8 38347200 PMC11001584

[B54] Darian-Smith I. Galea M. P. Darian-Smith C. Sugitani M. Tan A. Burman K. (1996). The anatomy of manual dexterity. The new connectivity of the primate sensorimotor thalamus and cerebral cortex. *Adv. Anat. Embryol. Cell Biol.* 133 1–140. 8854379

[B55] Darling W. G. Ge J. Stilwell-Morecraft K. S. Rotella D. L. Pizzimenti M. A. Morecraft R. J. (2018). Hand motor recovery following extensive frontoparietal cortical injury is accompanied by upregulated corticoreticular projections in monkey. *J. Neurosci.* 38 6323–6339. 10.1523/JNEUROSCI.0403-18.2018 29899028 PMC6041795

[B56] Dodd K. C. Nair V. A. Prabhakaran V. (2017). Role of the contralesional vs. ipsilesional hemisphere in stroke recovery. *Front. Hum. Neurosci.* 11:469. 10.3389/fnhum.2017.00469 28983244 PMC5613154

[B57] Du Beau A. Shakya Shrestha S. Bannatyne B. A. Jalicy S. M. Linnen S. Maxwell D. J. (2012). Neurotransmitter phenotypes of descending systems in the rat lumbar spinal cord. *Neuroscience* 227 67–79. 10.1016/j.neuroscience.2012.09.037 23018001

[B58] Ebbesen C. L. Brecht M. (2017). Motor cortex - to act or not to act? *Nat. Rev. Neurosci.* 18 694–705. 10.1038/nrn.2017.119 29042690

[B59] Esposito M. S. Capelli P. Arber S. (2014). Brainstem nucleus MdV mediates skilled forelimb motor tasks. *Nature* 508 351–356. 10.1038/nature13023 24487621

[B60] Fageiry S. Warriner C. L. Loper J. Pianski L. Reardon T. Jessell T. M. (2024). Distinct anatomical and functional corticospinal inputs innervate different spinal neuron types. *bioRxiv [Preprint]* 10.1101/2024.04.29.591683

[B61] Fait B. W. Cotto B. Murakami T. C. Hagemann-Jensen M. Zhan H. Freivald C. (2024). Spontaneously regenerative corticospinal neurons in mice. *bioRxiv [Preprint]* 10.1101/2024.09.09.612115 39314356 PMC11419066

[B62] Farr T. D. Whishaw I. Q. (2002). Quantitative and qualitative impairments in skilled reaching in the mouse (Mus musculus) after a focal motor cortex stroke. *Stroke* 33 1869–1875. 10.1161/01.str.0000020714.48349.4e 12105368

[B63] Fawcett J. W. (2009). Recovery from spinal cord injury: Regeneration, plasticity and rehabilitation. *Brain* 132 1417–1418. 10.1093/brain/awp121 19429905

[B64] Filli L. Engmann A. K. Zorner B. Weinmann O. Moraitis T. Gullo M. (2014). Bridging the gap: A reticulo-propriospinal detour bypassing an incomplete spinal cord injury. *J. Neurosci.* 34 13399–13410. 10.1523/JNEUROSCI.0701-14.2014 25274818 PMC6608315

[B65] Fink K. L. Cafferty W. B. (2016). Reorganization of intact descending motor circuits to replace lost connections after injury. *Neurotherapeutics* 13 370–381. 10.1007/s13311-016-0422-x 26846379 PMC4824020

[B66] Fink K. L. Lopez-Giraldez F. Kim I. J. Strittmatter S. M. Cafferty W. B. J. (2017). Identification of intrinsic axon growth modulators for intact CNS neurons after injury. *Cell Rep.* 18 2687–2701. 10.1016/j.celrep.2017.02.058 28297672 PMC5389739

[B67] Fouad K. Pedersen V. Schwab M. E. Brosamle C. (2001). Cervical sprouting of corticospinal fibers after thoracic spinal cord injury accompanies shifts in evoked motor responses. *Curr. Biol.* 11 1766–1770. 10.1016/s0960-9822(01)00535-8 11719218

[B68] Frezel N. Platonova E. Voigt F. F. Mateos J. M. Kastli R. Ziegler U. (2020). In-Depth characterization of layer 5 output neurons of the primary somatosensory cortex innervating the mouse dorsal spinal cord. *Cereb. Cortex Commun.* 1:tgaa052. 10.1093/texcom/tgaa052 34296117 PMC8152836

[B69] Friedli L. Rosenzweig E. S. Barraud Q. Schubert M. Dominici N. Awai L. (2015). Pronounced species divergence in corticospinal tract reorganization and functional recovery after lateralized spinal cord injury favors primates. *Sci. Transl. Med.* 7:302ra134. 10.1126/scitranslmed.aac5811 26311729 PMC5669362

[B70] Garcia-Alias G. Barkhuysen S. Buckle M. Fawcett J. W. (2009). Chondroitinase ABC treatment opens a window of opportunity for task-specific rehabilitation. *Nat. Neurosci.* 12 1145–1151. 10.1038/nn.2377 19668200

[B71] Garcia-Alias G. Truong K. Shah P. K. Roy R. R. Edgerton V. R. (2015). Plasticity of subcortical pathways promote recovery of skilled hand function in rats after corticospinal and rubrospinal tract injuries. *Exp. Neurol.* 266 112–119. 10.1016/j.expneurol.2015.01.009 25666586 PMC6952174

[B72] Geoffroy C. G. Zheng B. (2014). Myelin-associated inhibitors in axonal growth after CNS injury. *Curr. Opin. Neurobiol.* 27 31–38. 10.1016/j.conb.2014.02.012 24608164 PMC4122599

[B73] Ghosh A. Haiss F. Sydekum E. Schneider R. Gullo M. Wyss M. T. (2010). Rewiring of hindlimb corticospinal neurons after spinal cord injury. *Nat. Neurosci.* 13 97–104. 10.1038/nn.2448 20010824

[B74] Ghosh A. Sydekum E. Haiss F. Peduzzi S. Zorner B. Schneider R. (2009). Functional and anatomical reorganization of the sensory-motor cortex after incomplete spinal cord injury in adult rats. *J. Neurosci.* 29 12210–12219. 10.1523/JNEUROSCI.1828-09.2009 19793979 PMC6666156

[B75] Girgis J. Merrett D. Kirkland S. Metz G. A. Verge V. Fouad K. (2007). Reaching training in rats with spinal cord injury promotes plasticity and task specific recovery. *Brain* 130 2993–3003. 10.1093/brain/awm245 17928316

[B76] Goetz C. Pivetta C. Arber S. (2015). Distinct limb and trunk premotor circuits establish laterality in the spinal cord. *Neuron* 85 131–144. 10.1016/j.neuron.2014.11.024 25543457

[B77] Golan N. Ehrlich D. Bonanno J. O’Brien R. F. Murillo M. Kauer S. D. (2023). Anatomical diversity of the adult corticospinal tract revealed by single-cell transcriptional profiling. *J. Neurosci.* 43 7929–7945. 10.1523/JNEUROSCI.0811-22.2023 37748862 PMC10669816

[B78] Gomez-Pinilla F. Ying Z. Opazo P. Roy R. R. Edgerton V. R. (2001). Differential regulation by exercise of BDNF and NT-3 in rat spinal cord and skeletal muscle. *Eur. J. Neurosci.* 13 1078–1084. 10.1046/j.0953-816x.2001.01484.x 11285004

[B79] Goulding M. (2009). Circuits controlling vertebrate locomotion: Moving in a new direction. *Nat. Rev. Neurosci.* 10 507–518. 10.1038/nrn2608 19543221 PMC2847453

[B80] Gray P. A. (2013). Transcription factors define the neuroanatomical organization of the medullary reticular formation. *Front. Neuroanat.* 7:7. 10.3389/fnana.2013.00007 23717265 PMC3653110

[B81] Gu Z. Kalambogias J. Yoshioka S. Han W. Li Z. Kawasawa Y. I. (2017). Control of species-dependent cortico-motoneuronal connections underlying manual dexterity. *Science* 357, 400–404. 10.1126/science.aan3721 28751609 PMC5774341

[B82] Gu Z. Ueno M. Klinefelter K. Mamidi M. Yagi T. Yoshida Y. (2019). Skilled movements in mice require inhibition of corticospinal axon collateral formation in the spinal cord by semaphorin signaling. *J. Neurosci.* 39 8885–8899. 10.1523/JNEUROSCI.2832-18.2019 31537704 PMC6832677

[B83] Han Q. Cao C. Ding Y. So K. F. Wu W. Qu Y. (2015). Plasticity of motor network and function in the absence of corticospinal projection. *Exp. Neurol.* 267 194–208. 10.1016/j.expneurol.2015.03.008 25792481

[B84] Hata K. Fujitani M. Yasuda Y. Doya H. Saito T. Yamagishi S. (2006). RGMa inhibition promotes axonal growth and recovery after spinal cord injury. *J. Cell. Biol.* 173 47–58. 10.1083/jcb.200508143 16585268 PMC2063787

[B85] He Z. Jin Y. (2016). Intrinsic control of axon regeneration. *Neuron* 90 437–451. 10.1016/j.neuron.2016.04.022 27151637

[B86] Hermann G. E. Holmes G. M. Rogers R. C. Beattie M. S. Bresnahan J. C. (2003). Descending spinal projections from the rostral gigantocellular reticular nuclei complex. *J. Comp. Neurol.* 455 210–221. 10.1002/cne.10455 12454986

[B87] Hilton B. J. Anenberg E. Harrison T. C. Boyd J. D. Murphy T. H. Tetzlaff W. (2016). Re-establishment of cortical motor output maps and spontaneous functional recovery via spared dorsolaterally projecting corticospinal neurons after dorsal column spinal cord injury in adult mice. *J. Neurosci.* 36 4080–4092. 10.1523/JNEUROSCI.3386-15.2016 27053214 PMC6705513

[B88] Hollis E. R. (2016). Axon guidance molecules and neural circuit remodeling after spinal cord injury. *Neurotherapeutics* 13 360–369. 10.1007/s13311-015-0416-0 26676670 PMC4824017

[B89] Hollis E. R. Ishiko N. Yu T. Lu C. C. Haimovich A. Tolentino K. (2016). Ryk controls remapping of motor cortex during functional recovery after spinal cord injury. *Nat. Neurosci.* 19 697–705. 10.1038/nn.4282 27065364 PMC4847956

[B90] Holstege J. C. (1987). Brainstem projections to lumbar motoneurons in rat–II. An ultrastructural study by means of the anterograde transport of wheat germ agglutinin coupled to horseradish peroxidase and using the tetramethyl benzidine reaction. *Neuroscience* 21, 369–376. 10.1016/0306-4522(87)90127-8 2441313

[B91] Holstege J. C. (1991). Ultrastructural evidence for GABAergic brain stem projections to spinal motoneurons in the rat. *J. Neurosci.* 11, 159–167. 10.1523/JNEUROSCI.11-01-00159.1991 1702461 PMC6575203

[B92] Holstege J. C. Bongers C. M. (1991). A glycinergic projection from the ventromedial lower brainstem to spinal motoneurons. An ultrastructural double labeling study in rat. *Brain Res.* 566, 308–315. 10.1016/0006-8993(91)91715-d 1726063

[B93] Hossaini M. Goos J. A. Kohli S. K. Holstege J. C. (2012). Distribution of glycine/GABA neurons in the ventromedial medulla with descending spinal projections and evidence for an ascending glycine/GABA projection. *PLoS One* 7:e35293. 10.1371/journal.pone.0035293 22558137 PMC3340372

[B94] Huisman A. M. Kuypers H. G. Verburgh C. A. (1981). Quantitative differences in collateralization of the descending spinal pathways from red nucleus and other brain stem cell groups in rat as demonstrated with the multiple fluorescent retrograde tracer technique. *Brain Res.* 209 271–286. 10.1016/0006-8993(81)90153-0 7225794

[B95] Huisman A. M. Kuypers H. G. Conde F. Keizer K. (1983). Collaterals of rubrospinal neurons to the cerebellum in rat. A retrograde fluorescent double labeling study. *Brain Res.* 264 181–196. 10.1016/0006-8993(83)90816-8 6303500

[B96] Huma Z. Du Beau A. Brown C. Maxwell D. J. (2014). Origin and neurochemical properties of bulbospinal neurons projecting to the rat lumbar spinal cord via the medial longitudinal fasciculus and caudal ventrolateral medulla. *Front. Neural Circuits* 8:40. 10.3389/fncir.2014.00040 24808828 PMC4009430

[B97] Inoue T. Ikegami R. Takamatsu Y. Fukuchi M. Haga S. Ozaki M. (2023). Temporal dynamics of brain BDNF expression following a single bout of exercise: A bioluminescence imaging study. *Neurosci. Lett.* 799:137120. 10.1016/j.neulet.2023.137120 36764480

[B98] Inoue T. Okamura M. Kitahara M. Takamatsu Y. Sakakima H. Maejima H. (2020). Exercise plus pharmacological neuromodulation of synaptic inhibition enhance motor function recovery after ischemic stroke. *Neuroscience* 430 12–24. 10.1016/j.neuroscience.2020.01.012 31982464

[B99] Inoue T. Takamatsu Y. Nishio T. Soma K. Okamura M. Tohyama H. (2022). Combined treatment with exercise and alpha5GABAAR inhibitor promotes motor function recovery after intracerebral hemorrhage. *Neurosci. Lett.* 766:136344. 10.1016/j.neulet.2021.136344 34785309

[B100] Inoue T. Takamatsu Y. Okamura M. Maejima H. (2021). Ipsilateral BDNF mRNA expression in the motor cortex positively correlates with motor function of the affected forelimb after intracerebral hemorrhage. *Brain Res.* 1767 147536. 10.1016/j.brainres.2021.147536 34052261

[B101] Isa T. (2022). Double viral vector intersectional approaches for pathway-selective manipulation of motor functions and compensatory mechanisms. *Exp. Neurol.* 349:113959. 10.1016/j.expneurol.2021.113959 34953894

[B102] Isa T. Mitsuhashi M. Yamaguchi R. (2019). Alternative routes for recovery of hand functions after corticospinal tract injury in primates and rodents. *Curr. Opin. Neurol.* 32 836–843. 10.1097/WCO.0000000000000749 31688166

[B103] Isa T. Ohki Y. Alstermark B. Pettersson L. G. Sasaki S. (2007). Direct and indirect cortico-motoneuronal pathways and control of hand/arm movements. *Physiology (Bethesda)* 22 145–152. 10.1152/physiol.00045.2006 17420305

[B104] Ishida A. Isa K. Umeda T. Kobayashi K. Kobayashi K. Hida H. (2016). Causal link between the cortico-rubral pathway and functional recovery through forced impaired limb use in rats with stroke. *J. Neurosci.* 36 455–467. 10.1523/JNEUROSCI.2399-15.2016 26758837 PMC4710769

[B105] Ishida A. Kobayashi K. Ueda Y. Shimizu T. Tajiri N. Isa T. (2019). Dynamic Interaction between cortico-brainstem pathways during training-induced recovery in stroke model rats. *J. Neurosci.* 39 7306–7320. 10.1523/JNEUROSCI.0649-19.2019 31395620 PMC6759033

[B106] Jacobson P. B. Goody R. Lawrence M. Mueller B. K. Zhang X. Hooker B. A. (2021). Elezanumab, a human anti-RGMa monoclonal antibody, promotes neuroprotection, neuroplasticity, and neurorecovery following a thoracic hemicompression spinal cord injury in non-human primates. *Neurobiol. Dis.* 155:105385. 10.1016/j.nbd.2021.105385 33991647

[B107] Jarratt H. Hyland B. (1999). Neuronal activity in rat red nucleus during forelimb reach-to-grasp movements. *Neuroscience* 88 629–642. 10.1016/s0306-4522(98)00227-9 10197781

[B108] Jeffery N. D. Fitzgerald M. (2001). Effects of red nucleus ablation and exogenous neurotrophin-3 on corticospinal axon terminal distribution in the adult rat. *Neuroscience* 104 513–521. 10.1016/s0306-4522(01)00068-9 11377851

[B109] Jin D. Liu Y. Sun F. Wang X. Liu X. He Z. (2015). Restoration of skilled locomotion by sprouting corticospinal axons induced by co-deletion of PTEN and SOCS3. *Nat. Commun.* 6:8074. 10.1038/ncomms9074 26598325 PMC4662086

[B110] Jones B. E. Yang T. Z. (1985). The efferent projections from the reticular formation and the locus coeruleus studied by anterograde and retrograde axonal transport in the rat. *J. Comp. Neurol.* 242 56–92. 10.1002/cne.902420105 2416786

[B111] Jones T. A. (2017). Motor compensation and its effects on neural reorganization after stroke. *Nat. Rev. Neurosci.* 18 267–280. 10.1038/nrn.2017.26 28331232 PMC6289262

[B112] Jones T. A. Adkins D. L. (2015). Motor system reorganization after stroke: Stimulating and training toward perfection. *Physiology (Bethesda)* 30 358–370. 10.1152/physiol.00014.2015 26328881 PMC4556825

[B113] Jones T. A. Nemchek V. Fracassi M. (2024). Experience-driven competition in neural reorganization after stroke. *J. Physiol*. 603 737–757. 10.1113/JP285565 39476290 PMC11785499

[B114] Joy M. T. Carmichael S. T. (2021). Encouraging an excitable brain state: Mechanisms of brain repair in stroke. *Nat. Rev. Neurosci.* 22 38–53. 10.1038/s41583-020-00396-7 33184469 PMC10625167

[B115] Kaiser J. Maibach M. Salpeter I. Hagenbuch N. de Souza V. B. C. Robinson M. D. (2019). The spinal transcriptome after cortical stroke: In search of molecular factors regulating spontaneous recovery in the spinal cord. *J. Neurosci.* 39 4714–4726. 10.1523/JNEUROSCI.2571-18.2019 30962276 PMC6561692

[B116] Kameda H. Murabe N. Odagaki K. Mizukami H. Ozawa K. Sakurai M. (2019). Differential innervation within a transverse plane of spinal gray matter by sensorimotor cortices, with special reference to the somatosensory cortices. *J. Comp. Neurol.* 527 1401–1415. 10.1002/cne.24626 30620045

[B117] Kamiyama T. Kameda H. Murabe N. Fukuda S. Yoshioka N. Mizukami H. (2015). Corticospinal tract development and spinal cord innervation differ between cervical and lumbar targets. *J. Neurosci.* 35 1181–1191. 10.1523/JNEUROSCI.2842-13.2015 25609632 PMC6605536

[B118] Kanagal S. G. Muir G. D. (2008). Effects of combined dorsolateral and dorsal funicular lesions on sensorimotor behaviour in rats. *Exp. Neurol.* 214 229–239. 10.1016/j.expneurol.2008.08.004 18778707

[B119] Karadimas S. K. Satkunendrarajah K. Laliberte A. M. Ringuette D. Weisspapir I. Li L. (2020). Sensory cortical control of movement. *Nat. Neurosci.* 23 75–84. 10.1038/s41593-019-0536-7 31740813

[B120] Karbasforoushan H. Cohen-Adad J. Dewald J. P. A. (2019). Brainstem and spinal cord MRI identifies altered sensorimotor pathways post-stroke. *Nat. Commun.* 10:3524. 10.1038/s41467-019-11244-3 31388003 PMC6684621

[B121] Kathe C. Skinnider M. A. Hutson T. H. Regazzi N. Gautier M. Demesmaeker R. (2022). The neurons that restore walking after paralysis. *Nature* 611 540–547. 10.1038/s41586-022-05385-7 36352232 PMC9668750

[B122] Kinoshita M. Matsui R. Kato S. Hasegawa T. Kasahara H. Isa K. (2012). Genetic dissection of the circuit for hand dexterity in primates. *Nature* 487 235–238. 10.1038/nature11206 22722837

[B123] Kita T. Kita H. (2012). The subthalamic nucleus is one of multiple innervation sites for long-range corticofugal axons: A single-axon tracing study in the rat. *J. Neurosci.* 32 5990–5999. 10.1523/JNEUROSCI.5717-11.2012 22539859 PMC3479642

[B124] Klein A. Sacrey L. A. Whishaw I. Q. Dunnett S. B. (2012). The use of rodent skilled reaching as a translational model for investigating brain damage and disease. *Neurosci. Biobehav. Rev.* 36 1030–1042. 10.1016/j.neubiorev.2011.12.010 22227413

[B125] Kuang R. Z. Kalil K. (1990). Branching patterns of corticospinal axon arbors in the rodent. *J. Comp. Neurol.* 292 585–598. 10.1002/cne.902920408 2324314

[B126] Küchler M. Fouad K. Weinmann O. Schwab M. E. Raineteau O. (2002). Red nucleus projections to distinct motor neuron pools in the rat spinal cord. *J. Comp. Neurol.* 448 349–359. 10.1002/cne.10259 12115698

[B127] Kumamaru H. Lu P. Rosenzweig E. S. Kadoya K. Tuszynski M. H. (2019). Regenerating corticospinal axons innervate phenotypically appropriate neurons within neural stem cell grafts. *Cell Rep.* 26 2329–2339.e2324. 10.1016/j.celrep.2019.01.099 30811984 PMC6487864

[B128] Kumral E. Bayulkem G. Akyol A. Yunten N. Sirin H. Sagduyu A. (2002). Mesencephalic and associated posterior circulation infarcts. *Stroke* 33 2224–2231. 10.1161/01.str.0000027438.93029.87 12215591

[B129] Laliberte A. M. Goltash S. Lalonde N. R. Bui T. V. (2019). Propriospinal neurons: Essential elements of locomotor control in the intact and possibly the injured spinal cord. *Front. Cell. Neurosci.* 13:512. 10.3389/fncel.2019.00512 31798419 PMC6874159

[B130] Lapash Daniels C. M. Ayers K. L. Finley A. M. Culver J. P. Goldberg M. P. (2009). Axon sprouting in adult mouse spinal cord after motor cortex stroke. *Neurosci. Lett.* 450 191–195. 10.1016/j.neulet.2008.11.017 19022347 PMC2793320

[B131] Lee J. K. Geoffroy C. G. Chan A. F. Tolentino K. E. Crawford M. J. Leal M. A. (2010). Assessing spinal axon regeneration and sprouting in Nogo-, MAG-, and OMgp-deficient mice. *Neuron* 66 663–670. 10.1016/j.neuron.2010.05.002 20547125 PMC2896331

[B132] Lee J. K. Kim J. E. Sivula M. Strittmatter S. M. (2004). Nogo receptor antagonism promotes stroke recovery by enhancing axonal plasticity. *J. Neurosci.* 24 6209–6217. 10.1523/JNEUROSCI.1643-04.2004 15240813 PMC6729662

[B133] Lee K. H. Kim J. H. Choi D. H. Lee J. (2013). Effect of task-specific training on functional recovery and corticospinal tract plasticity after stroke. *Restor. Neurol. Neurosci.* 31 773–785. 10.3233/RNN-130336 24056148

[B134] Lee S. Ueno M. Yamashita T. (2011). Axonal remodeling for motor recovery after traumatic brain injury requires downregulation of gamma-aminobutyric acid signaling. *Cell Death Dis.* 2:e133. 10.1038/cddis.2011.16 21412279 PMC3101813

[B135] Leiras R. Cregg J. M. Kiehn O. (2022). Brainstem circuits for locomotion. *Annu. Rev. Neurosci.* 45 63–85. 10.1146/annurev-neuro-082321-025137 34985919

[B136] Lemon R. N. (2008). Descending pathways in motor control. *Annu. Rev. Neurosci.* 31 195–218. 10.1146/annurev.neuro.31.060407.125547 18558853

[B137] Liang F. Y. Moret V. Wiesendanger M. Rouiller E. M. (1991). Corticomotoneuronal connections in the rat: Evidence from double-labeling of motoneurons and corticospinal axon arborizations. *J. Comp. Neurol.* 311 356–366. 10.1002/cne.903110306 1720143

[B138] Liang H. Paxinos G. Watson C. (2011). Projections from the brain to the spinal cord in the mouse. *Brain Struct. Funct.* 215 159–186. 10.1007/s00429-010-0281-x 20936329

[B139] Liang H. Paxinos G. Watson C. (2012). The red nucleus and the rubrospinal projection in the mouse. *Brain Struct. Funct.* 217 221–232. 10.1007/s00429-011-0348-3 21927901

[B140] Liang H. Watson C. Paxinos G. (2015). Projections from the oral pontine reticular nucleus to the spinal cord of the mouse. *Neurosci. Lett.* 584 113–118. 10.1016/j.neulet.2014.10.025 25459287

[B141] Liang H. Watson C. Paxinos G. (2016). Terminations of reticulospinal fibers originating from the gigantocellular reticular formation in the mouse spinal cord. *Brain Struct. Funct.* 221 1623–1633. 10.1007/s00429-015-0993-z 25633472

[B142] Lie D. C. Song H. Colamarino S. A. Ming G. L. Gage F. H. (2004). Neurogenesis in the adult brain: New strategies for central nervous system diseases. *Annu. Rev. Pharmacol. Toxicol.* 44 399–421. 10.1146/annurev.pharmtox.44.101802.121631 14744252

[B143] Lin D. J. Cloutier A. M. Erler K. S. Cassidy J. M. Snider S. B. Ranford J. (2019). Corticospinal tract injury estimated from acute stroke imaging predicts upper extremity motor recovery after stroke. *Stroke* 50 3569–3577. 10.1161/STROKEAHA.119.025898 31648631 PMC6878199

[B144] Lindau N. T. Banninger B. J. Gullo M. Good N. A. Bachmann L. C. Starkey M. L. (2014). Rewiring of the corticospinal tract in the adult rat after unilateral stroke and anti-Nogo-A therapy. *Brain* 137 739–756. 10.1093/brain/awt336 24355710

[B145] Liu B. Li L. L. Tan X. D. Zhang Y. H. Jiang Y. He G. Q. (2015). Gadd45b mediates axonal plasticity and subsequent functional recovery after experimental stroke in rats. *Mol. Neurobiol.* 52 1245–1256. 10.1007/s12035-014-8909-0 25324012

[B146] Liu K. Lu Y. Lee J. K. Samara R. Willenberg R. Sears-Kraxberger I. (2010). PTEN deletion enhances the regenerative ability of adult corticospinal neurons. *Nat. Neurosci.* 13 1075–1081. 10.1038/nn.2603 20694004 PMC2928871

[B147] Liu K. Tedeschi A. Park K. K. He Z. (2011). Neuronal intrinsic mechanisms of axon regeneration. *Annu. Rev. Neurosci.* 34 131–152. 10.1146/annurev-neuro-061010-113723 21438684

[B148] Liu Y. Latremoliere A. Li X. Zhang Z. Chen M. Wang X. (2018a). Touch and tactile neuropathic pain sensitivity are set by corticospinal projections. *Nature* 561 547–550. 10.1038/s41586-018-0515-2 30209395 PMC6163083

[B149] Liu Y. Lu G. Su X. W. Ding T. Wang W. L. Li Y. M. (2018b). Characterization of axon damage, neurological deficits, and histopathology in two experimental models of intracerebral hemorrhage. *Front. Neurosci.* 12:928. 10.3389/fnins.2018.00928 30618557 PMC6297275

[B150] Liu Z. Li Y. Zhang X. Savant-Bhonsale S. Chopp M. (2008). Contralesional axonal remodeling of the corticospinal system in adult rats after stroke and bone marrow stromal cell treatment. *Stroke* 39 2571–2577. 10.1161/STROKEAHA.107.511659 18617661 PMC2593106

[B151] Lopez-Virgen V. Olivares-Moreno R. de Lafuente V. Concha L. Rojas-Piloni G. (2022). Different subtypes of motor cortex pyramidal tract neurons projects to red and pontine nuclei. *Front. Cell. Neurosci.* 16:1073731. 10.3389/fncel.2022.1073731 36605617 PMC9807917

[B152] Low A. Y. T. Thanawalla A. R. Yip A. K. K. Kim J. Wong K. L. L. Tantra M. (2018). Precision of discrete and rhythmic forelimb movements requires a distinct neuronal subpopulation in the interposed anterior nucleus. *Cell Rep.* 22 2322–2333. 10.1016/j.celrep.2018.02.017 29490269

[B153] Lu P. Blesch A. Graham L. Wang Y. Samara R. Banos K. (2012). Motor axonal regeneration after partial and complete spinal cord transection. *J. Neurosci.* 32 8208–8218. 10.1523/JNEUROSCI.0308-12.2012 22699902 PMC3407545

[B154] Lu T. Shinozaki M. Nagoshi N. Nakamura M. Okano H. (2022). 3D imaging of supraspinal inputs to the thoracic and lumbar spinal cord mapped by retrograde tracing and light-sheet microscopy. *J. Neurochem.* 162 352–370. 10.1111/jnc.15653 35674500

[B155] Luo L. Callaway E. M. Svoboda K. (2018). Genetic dissection of neural circuits: A decade of progress. *Neuron* 98 256–281. 10.1016/j.neuron.2018.03.040 29673479 PMC5912347

[B156] Maeda H. Fukuda S. Kameda H. Murabe N. Isoo N. Mizukami H. (2016). Corticospinal axons make direct synaptic connections with spinal motoneurons innervating forearm muscles early during postnatal development in the rat. *J. Physiol.* 594 189–205. 10.1113/JP270885 26503304 PMC4704511

[B157] Mahar M. Cavalli V. (2018). Intrinsic mechanisms of neuronal axon regeneration. *Nat. Rev. Neurosci.* 19 323–337. 10.1038/s41583-018-0001-8 29666508 PMC5987780

[B158] Maier I. C. Baumann K. Thallmair M. Weinmann O. Scholl J. Schwab M. E. (2008). Constraint-induced movement therapy in the adult rat after unilateral corticospinal tract injury. *J. Neurosci.* 28 9386–9403. 10.1523/JNEUROSCI.1697-08.2008 18799672 PMC6671131

[B159] Maier I. C. Ichiyama R. M. Courtine G. Schnell L. Lavrov I. Edgerton V. R. (2009). Differential effects of anti-Nogo-A antibody treatment and treadmill training in rats with incomplete spinal cord injury. *Brain* 132 1426–1440. 10.1093/brain/awp085 19372269

[B160] Martin G. F. Vertes R. P. Waltzer R. (1985). Spinal projections of the gigantocellular reticular formation in the rat. Evidence for projections from different areas to laminae I and II and lamina IX. *Exp. Brain Res.* 58 154–162. 10.1007/BF00238963 3987846

[B161] Massion J. (1967). The mammalian red nucleus. *Physiol. Rev.* 47 383–436. 10.1152/physrev.1967.47.3.383 4864540

[B162] Massion J. (1988). Red nucleus: Past and future. *Behav. Brain Res.* 28 1–8. 10.1016/0166-4328(88)90071-x 3289561

[B163] Matsumoto N. Barson D. Liang L. Crair M. C. (2024). Hebbian instruction of axonal connectivity by endogenous correlated spontaneous activity. *Science* 385:eadh7814. 10.1126/science.adh7814 39146415 PMC12148345

[B164] Maynard G. Kannan R. Liu J. Wang W. Lam T. K. T. Wang X. (2023). Soluble Nogo-Receptor-Fc decoy (AXER-204) in patients with chronic cervical spinal cord injury in the USA: A first-in-human and randomised clinical trial. *Lancet Neurol.* 22 672–684. 10.1016/S1474-4422(23)00215-6 37479373 PMC10410101

[B165] Miller M. W. (1987). The origin of corticospinal projection neurons in rat. *Exp. Brain Res.* 67 339–351. 10.1007/BF00248554 3622693

[B166] Minnerup J. Strecker J. K. Wachsmuth L. Hoppen M. Schmidt A. Hermann D. M. (2018). Defining mechanisms of neural plasticity after brainstem ischemia in rats. *Ann. Neurol.* 83 1003–1015. 10.1002/ana.25238 29665155

[B167] Mitchell E. J. McCallum S. Dewar D. Maxwell D. J. (2016). Corticospinal and reticulospinal contacts on cervical commissural and long descending propriospinal neurons in the adult rat spinal cord; Evidence for powerful reticulospinal connections. *PLoS One* 11:e0152094. 10.1371/journal.pone.0152094 26999665 PMC4801400

[B168] Morecraft R. J. Ge J. Stilwell-Morecraft K. S. McNeal D. W. Hynes S. M. Pizzimenti M. A. (2016). Frontal and frontoparietal injury differentially affect the ipsilateral corticospinal projection from the nonlesioned hemisphere in monkey (*Macaca mulatta*). *J. Comp. Neurol.* 524 380–407. 10.1002/cne.23861 26224429 PMC4675697

[B169] Moreno-Lopez Y. Bichara C. Delbecq G. Isope P. Cordero-Erausquin M. (2021). The corticospinal tract primarily modulates sensory inputs in the mouse lumbar cord. *Elife* 10:e65304. 10.7554/eLife.65304 34497004 PMC8439650

[B170] Morris R. Whishaw I. Q. (2016). A proposal for a rat model of spinal cord injury featuring the rubrospinal tract and its contributions to locomotion and skilled hand movement. *Front. Neurosci.* 10:5. 10.3389/fnins.2016.00005 26858587 PMC4728831

[B171] Morris R. Tosolini A. P. Goldstein J. D. Whishaw I. Q. (2011). Impaired arpeggio movement in skilled reaching by rubrospinal tract lesions in the rat: A behavioral/anatomical fractionation. *J. Neurotrauma* 28 2439–2451. 10.1089/neu.2010.1708 21612320

[B172] Morris R. Vallester K. K. Newton S. S. Kearsley A. P. Whishaw I. Q. (2015). The differential contributions of the parvocellular and the magnocellular subdivisions of the red nucleus to skilled reaching in the rat. *Neuroscience* 295 48–57. 10.1016/j.neuroscience.2015.03.027 25813707

[B173] Mosberger A. C. Miehlbradt J. C. Bjelopoljak N. Schneider M. P. Wahl A. S. Ineichen B. V. (2018). Axotomized corticospinal neurons increase supra-lesional innervation and remain crucial for skilled reaching after bilateral pyramidotomy. *Cereb. Cortex* 28 625–643. 10.1093/cercor/bhw405 28069760

[B174] Muir G. D. Whishaw I. Q. (2000). Red nucleus lesions impair overground locomotion in rats: A kinetic analysis. *Eur J. Neurosci.* 12 1113–1122. 10.1046/j.1460-9568.2000.00987.x 10762342

[B175] Murphy T. H. Corbett D. (2009). Plasticity during stroke recovery: From synapse to behaviour. *Nat. Rev. Neurosci.* 10 861–872. 10.1038/nrn2735 19888284

[B176] Murray A. J. Croce K. Belton T. Akay T. Jessell T. M. (2018). Balance control mediated by vestibular circuits directing limb extension or antagonist muscle co-activation. *Cell Rep.* 22 1325–1338. 10.1016/j.celrep.2018.01.009 29386118

[B177] Murray H. M. Gurule M. E. (1979). Origin of the rubrospinal tract of the rat. *Neurosci. Lett.* 14 19–23. 10.1016/0304-3940(79)95337-0 530486

[B178] Nakagawa H. Ninomiya T. Yamashita T. Takada M. (2019). Treatment with the neutralizing antibody against repulsive guidance molecule-a promotes recovery from impaired manual dexterity in a primate model of spinal cord injury. *Cereb. Cortex* 29 561–572. 10.1093/cercor/bhx338 29315368

[B179] Nakagawa H. Ueno M. Itokazu T. Yamashita T. (2013). Bilateral movement training promotes axonal remodeling of the corticospinal tract and recovery of motor function following traumatic brain injury in mice. *Cell Death Dis.* 4:e534. 10.1038/cddis.2013.62 23470541 PMC3613840

[B180] Nakamura Y. Ueno M. Niehaus J. K. Lang R. A. Zheng Y. Yoshida Y. (2021). Modulation of both intrinsic and extrinsic factors additively promotes rewiring of corticospinal circuits after spinal cord injury. *J. Neurosci.* 41 10247–10260. 10.1523/JNEUROSCI.2649-20.2021 34759029 PMC8672694

[B181] Napieralski J. A. Butler A. K. Chesselet M. F. (1996). Anatomical and functional evidence for lesion-specific sprouting of corticostriatal input in the adult rat. *J. Comp. Neurol.* 373 484–497. 8889940 10.1002/(SICI)1096-9861(19960930)373:4<484::AID-CNE2>3.0.CO;2-Y

[B182] Nelson A. Abdelmesih B. Costa R. M. (2021). Corticospinal populations broadcast complex motor signals to coordinated spinal and striatal circuits. *Nat. Neurosci.* 24 1721–1732. 10.1038/s41593-021-00939-w 34737448 PMC8639707

[B183] Ni Y. Nawabi H. Liu X. Yang L. Miyamichi K. Tedeschi A. (2014). Characterization of long descending premotor propriospinal neurons in the spinal cord. *J. Neurosci.* 34 9404–9417. 10.1523/JNEUROSCI.1771-14.2014 25009272 PMC4468139

[B184] Nicola F. D. C. Hua I. Levine A. J. (2022). Intersectional genetic tools to study skilled reaching in mice. *Exp. Neurol.* 347:113879. 10.1016/j.expneurol.2021.113879 34597682

[B185] Nishibe M. Barbay S. Guggenmos D. Nudo R. J. (2010). Reorganization of motor cortex after controlled cortical impact in rats and implications for functional recovery. *J. Neurotrauma* 27 2221–2232. 10.1089/neu.2010.1456 20873958 PMC2996815

[B186] Nishibe M. Urban E. T. Barbay S. Nudo R. J. (2015). Rehabilitative training promotes rapid motor recovery but delayed motor map reorganization in a rat cortical ischemic infarct model. *Neurorehabil. Neural Repair.* 29 472–482. 10.1177/1545968314543499 25055836 PMC4303553

[B187] Nudo R. J. Masterton R. B. (1988). Descending pathways to the spinal cord: A comparative study of 22 mammals. *J. Comp. Neurol.* 277 53–79. 10.1002/cne.902770105 3198796

[B188] Okabe N. Miyamoto O. (2018). Role and limitations of rehabilitation-induced neural network remodeling after stroke. *Neural Regen. Res.* 13 2087–2088. 10.4103/1673-5374.241450 30323129 PMC6199934

[B189] Okabe N. Himi N. Maruyama-Nakamura E. Hayashi N. Narita K. Miyamoto O. (2017a). Rehabilitative skilled forelimb training enhances axonal remodeling in the corticospinal pathway but not the brainstem-spinal pathways after photothrombotic stroke in the primary motor cortex. *PLoS One* 12:e0187413. 10.1371/journal.pone.0187413 29095902 PMC5667818

[B190] Okabe N. Himi N. Nakamura-Maruyama E. Hayashi N. Sakamoto I. Narita K. (2018). Constraint-induced movement therapy improves efficacy of task-specific training after severe cortical stroke depending on the ipsilesional corticospinal projections. *Exp. Neurol.* 305 108–120. 10.1016/j.expneurol.2018.04.006 29653186

[B191] Okabe N. Narita K. Miyamoto O. (2017b). Axonal remodeling in the corticospinal tract after stroke: How does rehabilitative training modulate it? *Neural Regen. Res.* 12 185–192. 10.4103/1673-5374.200792 28400791 PMC5361493

[B192] Okabe N. Shiromoto T. Himi N. Lu F. Maruyama-Nakamura E. Narita K. (2016). Neural network remodeling underlying motor map reorganization induced by rehabilitative training after ischemic stroke. *Neuroscience* 339 338–362. 10.1016/j.neuroscience.2016.10.008 27725217

[B193] Olivares-Moreno R. Rodriguez-Moreno P. Lopez-Virgen V. Macias M. Altamira-Camacho M. Rojas-Piloni G. (2021). Corticospinal vs rubrospinal revisited: An evolutionary perspective for sensorimotor integration. *Front. Neurosci.* 15:686481. 10.3389/fnins.2021.686481 34177458 PMC8226017

[B194] Omoto S. Ueno M. Mochio S. Yamashita T. (2011). Corticospinal tract fibers cross the ephrin-B3-negative part of the midline of the spinal cord after brain injury. *Neurosci. Res.* 69 187–195. 10.1016/j.neures.2010.12.004 21147179

[B195] Omoto S. Ueno M. Mochio S. Takai T. Yamashita T. (2010). Genetic deletion of paired immunoglobulin-like receptor B does not promote axonal plasticity or functional recovery after traumatic brain injury. *J. Neurosci.* 30 13045–13052. 10.1523/JNEUROSCI.3228-10.2010 20881122 PMC6633514

[B196] Osseward P. J. Amin N. D. Moore J. D. Temple B. A. Barriga B. K. Bachmann L. C. (2021). Conserved genetic signatures parcellate cardinal spinal neuron classes into local and projection subsets. *Science* 372 385–393. 10.1126/science.abe0690 33888637 PMC8612134

[B197] Papadopoulos C. M. Tsai S. Y. Alsbiei T. O’Brien T. E. Schwab M. E. Kartje G. L. (2002). Functional recovery and neuroanatomical plasticity following middle cerebral artery occlusion and IN-1 antibody treatment in the adult rat. *Ann. Neurol.* 51 433–441. 10.1002/ana.10144 11921049

[B198] Perreault M. C. Giorgi A. (2019). Diversity of reticulospinal systems in mammals. *Curr. Opin. Physiol.* 8 161–169. 10.1016/j.cophys.2019.03.001 31763514 PMC6874378

[B199] Perreault M. C. Glover J. C. (2013). Glutamatergic reticulospinal neurons in the mouse: Developmental origins, axon projections, and functional connectivity. *Ann. N. Y. Acad. Sci.* 1279 80–89. 10.1111/nyas.12054 23531005

[B200] Pourchet O. Morel M. P. Welniarz Q. Sarrazin N. Marti F. Heck N. (2021). Loss of floor plate Netrin-1 impairs midline crossing of corticospinal axons and leads to mirror movements. *Cell Rep.* 34:108654. 10.1016/j.celrep.2020.108654 33472083

[B201] Raineteau O. Schwab M. E. (2001). Plasticity of motor systems after incomplete spinal cord injury. *Nat. Rev. Neurosci.* 2 263–273. 10.1038/35067570 11283749

[B202] Raineteau O. Fouad K. Bareyre F. M. Schwab M. E. (2002). Reorganization of descending motor tracts in the rat spinal cord. *Eur. J. Neurosci.* 16 1761–1771. 10.1046/j.1460-9568.2002.02243.x 12431229

[B203] Raineteau O. Fouad K. Noth P. Thallmair M. Schwab M. E. (2001). Functional switch between motor tracts in the presence of the mAb IN-1 in the adult rat. *Proc. Natl. Acad. Sci. U S A.* 98 6929–6934. 10.1073/pnas.111165498 11381120 PMC34455

[B204] Reed W. R. Shum-Siu A. Magnuson D. S. (2008). Reticulospinal pathways in the ventrolateral funiculus with terminations in the cervical and lumbar enlargements of the adult rat spinal cord. *Neuroscience* 151 505–517. 10.1016/j.neuroscience.2007.10.025 18065156 PMC2829753

[B205] Reid J. M. Gwym D. G. Flumerfelt B. A. (1975). A cytoarchitectonic and Golgi study of the red nucleus in the rat. *J. Comp. Neurol.* 162 337–361. 10.1002/cne.901620305 50332

[B206] Reinoß P. Ciglieri E. Minere M. Bremser S. Klein A. Lohr H. (2020). Hypothalamic Pomc neurons innervate the spinal cord and modulate the excitability of premotor circuits. *Curr. Biol.* 30 4579–4593.e4577. 10.1016/j.cub.2020.08 32976803 PMC8847999

[B207] Rizzi G. Coban M. Tan K. R. (2019). Excitatory rubral cells encode the acquisition of novel complex motor tasks. *Nat. Commun.* 10:2241. 10.1038/s41467-019-10223-y 31113944 PMC6529416

[B208] Rouiller E. M. Liang F. Y. Moret V. Wiesendanger M. (1991). Trajectory of redirected corticospinal axons after unilateral lesion of the sensorimotor cortex in neonatal rat; A phaseolus vulgaris-leucoagglutinin (PHA-L) tracing study. *Exp. Neurol.* 114 53–65. 10.1016/0014-4886(91)90084-p 1915735

[B209] Ruber T. Schlaug G. Lindenberg R. (2012). Compensatory role of the cortico-rubro-spinal tract in motor recovery after stroke. *Neurology* 79 515–522. 10.1212/WNL.0b013e31826356e8 22843266 PMC3413760

[B210] Ruder L. Arber S. (2019). Brainstem circuits controlling action diversification. *Annu. Rev. Neurosci.* 42 485–504. 10.1146/annurev-neuro-070918-050201 31283898

[B211] Ruder L. Schina R. Kanodia H. Valencia-Garcia S. Pivetta C. Arber S. (2021). A functional map for diverse forelimb actions within brainstem circuitry. *Nature* 590 445–450. 10.1038/s41586-020-03080-z 33408409

[B212] Ruder L. Takeoka A. Arber S. (2016). Long-distance descending spinal neurons ensure quadrupedal locomotor stability. *Neuron* 92 1063–1078. 10.1016/j.neuron.2016.10.032 27866798

[B213] Ruigrok T. J. Pijpers A. Goedknegt-Sabel E. Coulon P. (2008). Multiple cerebellar zones are involved in the control of individual muscles: A retrograde transneuronal tracing study with rabies virus in the rat. *Eur. J. Neurosci.* 28 181–200. 10.1111/j.1460-9568.2008.06294.x 18662342

[B214] Russ D. E. Cross R. B. P. Li L. Koch S. C. Matson K. J. E. Yadav A. (2021). A harmonized atlas of mouse spinal cord cell types and their spatial organization. *Nat. Commun.* 12:5722. 10.1038/s41467-021-25125-1 34588430 PMC8481483

[B215] Sacrey L. A. Alaverdashvili M. Whishaw I. Q. (2009). Similar hand shaping in reaching-for-food (skilled reaching) in rats and humans provides evidence of homology in release, collection, and manipulation movements. *Behav. Brain Res.* 204 153–161. 10.1016/j.bbr.2009.05.035 19520119

[B216] Sahni V. Itoh Y. Shnider S. J. Macklis J. D. (2021a). Crim1 and Kelch-like 14 exert complementary dual-directional developmental control over segmentally specific corticospinal axon projection targeting. *Cell. Rep.* 37:109842. 10.1016/j.celrep.2021.109842 34686337 PMC8697027

[B217] Sahni V. Shnider S. J. Jabaudon D. Song J. H. T. Itoh Y. Greig L. C. (2021b). Corticospinal neuron subpopulation-specific developmental genes prospectively indicate mature segmentally specific axon projection targeting. *Cell Rep.* 37:109843. 10.1016/j.celrep.2021.109843 34686320 PMC8653526

[B218] Sathyamurthy A. Barik A. Dobrott C. I. Matson K. J. E. Stoica S. Pursley R. (2020). Cerebellospinal neurons regulate motor performance and motor learning. *Cell Rep.* 31:107595. 10.1016/j.celrep.2020.107595 32402292 PMC7263484

[B219] Sato T. Nakamura Y. Hoshina K. Inoue K.-I. Takada M. Yano M. (2025). Scg2 drives reorganization of the corticospinal circuit with spinal premotor interneurons to recover motor function after stroke. *bioRxiv [Preprint]* 10.1101/2025.01.21.634186 PMC13249867 42265101

[B220] Sato T. Nakamura Y. Takeda A. Ueno M. (2021). Lesion area in the cerebral cortex determines the patterns of axon rewiring of motor and sensory corticospinal tracts after stroke. *Front. Neurosci.* 15:737034. 10.3389/fnins.2021.737034 34707476 PMC8542932

[B221] Schwarz L. A. Miyamichi K. Gao X. J. Beier K. T. Weissbourd B. DeLoach K. E. (2015). Viral-genetic tracing of the input-output organization of a central noradrenaline circuit. *Nature* 524 88–92. 10.1038/nature14600 26131933 PMC4587569

[B222] Seo J. P. Jang S. H. (2013). Different characteristics of the corticospinal tract according to the cerebral origin: DTI study. *AJNR Am. J. Neuroradiol.* 34 1359–1363. 10.3174/ajnr.A3389 23370470 PMC8051479

[B223] Serradj N. Marino F. Moreno-Lopez Y. Bernstein A. Agger S. Soliman M. (2023). Task-specific modulation of corticospinal neuron activity during motor learning in mice. *Nat. Commun.* 14:2708. 10.1038/s41467-023-38418-4 37169765 PMC10175564

[B224] Sheikh I. S. Keefe K. M. Sterling N. A. Junker I. P. Li C. Chen J. (2024). Compensatory adaptation of parallel motor pathways promotes skilled forelimb recovery after spinal cord injury. *iScience* 27:111371. 10.1016/j.isci.2024.111371 39654633 PMC11626773

[B225] Shieh J. Y. Leong S. K. Wong W. C. (1983). Origin of the rubrospinal tract in neonatal, developing, and mature rats. *J. Comp. Neurol.* 214 79–86. 10.1002/cne.902140108 6841678

[B226] Siegel C. S. Fink K. L. Strittmatter S. M. Cafferty W. B. (2015). Plasticity of intact rubral projections mediates spontaneous recovery of function after corticospinal tract injury. *J. Neurosci.* 35 1443–1457. 10.1523/JNEUROSCI.3713-14.2015 25632122 PMC4308593

[B227] Silver J. Schwab M. E. Popovich P. G. (2014). Central nervous system regenerative failure: Role of oligodendrocytes, astrocytes, and microglia. *Cold Spring Harb. Perspect. Biol.* 7:a020602. 10.1101/cshperspect.a020602 25475091 PMC4355267

[B228] Sinopoulou E. Rosenzweig E. S. Conner J. M. Gibbs D. Weinholtz C. A. Weber J. L. (2022). Rhesus macaque versus rat divergence in the corticospinal projectome. *Neuron* 110 2970–2983.e2974. 10.1016/j.neuron.2022.07.002 35917818 PMC9509478

[B229] Sivertsen M. S. Perreault M. C. Glover J. C. (2016). Pontine reticulospinal projections in the neonatal mouse: Internal organization and axon trajectories. *J. Comp. Neurol.* 524 1270–1291. 10.1002/cne.23904 26400815 PMC4851107

[B230] Sooksawate T. Isa K. Matsui R. Kato S. Kinoshita M. Kobayashi K. (2013). Viral vector-mediated selective and reversible blockade of the pathway for visual orienting in mice. *Front. Neural Circuits* 7:162. 10.3389/fncir.2013.00162 24130520 PMC3795302

[B231] Squair J. W. Milano M. de Coucy A. Gautier M. Skinnider M. A. James N. D. (2023). Recovery of walking after paralysis by regenerating characterized neurons to their natural target region. *Science* 381 1338–1345. 10.1126/science.adi6412 37733871

[B232] Starkey M. L. Barritt A. W. Yip P. K. Davies M. Hamers F. P. McMahon S. B. (2005). Assessing behavioural function following a pyramidotomy lesion of the corticospinal tract in adult mice. *Exp. Neurol.* 195 524–539. 10.1016/j.expneurol.2005.06.017 16051217

[B233] Starkey M. L. Bartus K. Barritt A. W. Bradbury E. J. (2012a). Chondroitinase ABC promotes compensatory sprouting of the intact corticospinal tract and recovery of forelimb function following unilateral pyramidotomy in adult mice. *Eur. J. Neurosci.* 36 3665–3678. 10.1111/ejn.12017 23061434 PMC4851235

[B234] Starkey M. L. Bleul C. Zorner B. Lindau N. T. Mueggler T. Rudin M. (2012b). Back seat driving: Hindlimb corticospinal neurons assume forelimb control following ischaemic stroke. *Brain* 135 3265–3281. 10.1093/brain/aws270 23169918

[B235] Stepien A. E. Tripodi M. Arber S. (2010). Monosynaptic rabies virus reveals premotor network organization and synaptic specificity of cholinergic partition cells. *Neuron* 68 456–472. 10.1016/j.neuron.2010.10.019 21040847

[B236] Steward O. Yee K. M. Metcalfe M. Willenberg R. Luo J. Azevedo R. (2021). Rostro-caudal specificity of corticospinal tract projections in mice. *Cereb. Cortex* 31 2322–2344. 10.1093/cercor/bhaa338 33350438 PMC8023844

[B237] Strominger R. N. McGiffen J. E. Strominger N. L. (1987). Morphometric and experimental studies of the red nucleus in the albino rat. *Anat. Rec.* 219 420–428. 10.1002/ar.1092190413 3448957

[B238] Suzuki K. Elegheert J. Song I. Sasakura H. Senkov O. Matsuda K. (2020). A synthetic synaptic organizer protein restores glutamatergic neuronal circuits. *Science* 369:abb4853. 10.1126/science.abb4853 32855309 PMC7116145

[B239] Swenson R. S. Castro A. J. (1983). The afferent connections of the inferior olivary complex in rats. An anterograde study using autoradiographic and axonal degeneration techniques. *Neuroscience* 8 259–275. 10.1016/0306-4522(83)90064-7 6843823

[B240] Szokol K. Glover J. C. Perreault M. C. (2008). Differential origin of reticulospinal drive to motoneurons innervating trunk and hindlimb muscles in the mouse revealed by optical recording. *J. Physiol.* 586 5259–5276. 10.1113/jphysiol.2008.158105 18772205 PMC2652148

[B241] Szokol K. Glover J. C. Perreault M. C. (2011). Organization of functional synaptic connections between medullary reticulospinal neurons and lumbar descending commissural interneurons in the neonatal mouse. *J. Neurosci.* 31 4731–4742. 10.1523/JNEUROSCI.5486-10.2011 21430172 PMC6622908

[B242] Takenobu Y. Hayashi T. Moriwaki H. Nagatsuka K. Naritomi H. Fukuyama H. (2014). Motor recovery and microstructural change in rubro-spinal tract in subcortical stroke. *Neuroimage Clin.* 4 201–208. 10.1016/j.nicl.2013.12.003 24432247 PMC3891492

[B243] Tan A. M. Samad O. A. Fischer T. Z. Zhao P. Persson A. K. Waxman S. G. (2012). Maladaptive dendritic spine remodeling contributes to diabetic neuropathic pain. *J. Neurosci.* 32 6795–6807. 10.1523/JNEUROSCI.1017-12.2012 22593049 PMC6622192

[B244] Tanaka T. Ito T. Sumizono M. Ono M. Kato N. Honma S. (2020). Combinational approach of genetic SHP-1 suppression and voluntary exercise promotes corticospinal tract sprouting and motor recovery following brain injury. *Neurorehabil. Neural Repair.* 34 558–570. 10.1177/1545968320921827 32441214

[B245] Tennant K. A. Adkins D. L. Donlan N. A. Asay A. L. Thomas N. Kleim J. A. (2011). The organization of the forelimb representation of the C57BL/6 mouse motor cortex as defined by intracortical microstimulation and cytoarchitecture. *Cereb. Cortex* 21 865–876. 10.1093/cercor/bhq159 20739477 PMC3059888

[B246] Thallmair M. Metz G. A. Z’Graggen W. J. Raineteau O. Kartje G. L. Schwab M. E. (1998). Neurite growth inhibitors restrict plasticity and functional recovery following corticospinal tract lesions. *Nat. Neurosci.* 1 124–131. 10.1038/373 10195127

[B247] Tuszynski M. H. Steward O. (2012). Concepts and methods for the study of axonal regeneration in the CNS. *Neuron* 74 777–791. 10.1016/j.neuron.2012.05.006 22681683 PMC3387806

[B248] Ueno M. Hayano Y. Nakagawa H. Yamashita T. (2012). Intraspinal rewiring of the corticospinal tract requires target-derived brain-derived neurotrophic factor and compensates lost function after brain injury. *Brain* 135 1253–1267. 10.1093/brain/aws053 22436236

[B249] Ueno M. Nakamura Y. Li J. Gu Z. Niehaus J. Maezawa M. (2018). Corticospinal circuits from the sensory and motor cortices differentially regulate skilled movements through distinct spinal interneurons. *Cell Rep.* 23 1286–1300.e1287. 10.1016/j.celrep.2018.03.137 29719245 PMC6608728

[B250] Ueno M. Nakamura Y. Nakagawa H. Niehaus J. K. Maezawa M. Gu Z. (2020). Olig2-induced semaphorin expression drives corticospinal axon retraction after spinal cord injury. *Cereb. Cortex* 30 5702–5716. 10.1093/cercor/bhaa142 32564090 PMC8179623

[B251] Usseglio G. Gatier E. Heuze A. Herent C. Bouvier J. (2020). Control of orienting movements and locomotion by projection-defined subsets of brainstem V2a neurons. *Curr. Biol.* 30 4665–4681.e4666. 10.1016/j.cub.2020.09.014 33007251

[B252] Usuda N. Sugawara S. K. Fukuyama H. Nakazawa K. Amemiya K. Nishimura Y. (2022). Quantitative comparison of corticospinal tracts arising from different cortical areas in humans. *Neurosci. Res.* 183 30–49. 10.1016/j.neures.2022.06.008 35787428

[B253] Valencia Garcia S. Brischoux F. Clement O. Libourel P. A. Arthaud S. Lazarus M. (2018). Ventromedial medulla inhibitory neuron inactivation induces REM sleep without atonia and REM sleep behavior disorder. *Nat. Commun.* 9:504. 10.1038/s41467-017-02761-0 29402935 PMC5799338

[B254] van den Brand R. Heutschi J. Barraud Q. DiGiovanna J. Bartholdi K. Huerlimann M. (2012). Restoring voluntary control of locomotion after paralyzing spinal cord injury. *Science* 336 1182–1185. 10.1126/science.1217416 22654062

[B255] Van Steenbergen V. Burattini L. Trumpp M. Fourneau J. Aljovic A. Chahin M. (2023). Coordinated neurostimulation promotes circuit rewiring and unlocks recovery after spinal cord injury. *J. Exp. Med.* 220:e20220615. 10.1084/jem.20220615 36571760 PMC9794600

[B256] VanderHorst V. G. Ulfhake B. (2006). The organization of the brainstem and spinal cord of the mouse: Relationships between monoaminergic, cholinergic, and spinal projection systems. *J. Chem. Neuroanat.* 31 2–36. 10.1016/j.jchemneu.2005.08.003 16183250

[B257] Vavrek R. Girgis J. Tetzlaff W. Hiebert G. W. Fouad K. (2006). BDNF promotes connections of corticospinal neurons onto spared descending interneurons in spinal cord injured rats. *Brain* 129 1534–1545. 10.1093/brain/awl087 16632552

[B258] Wahl A. S. Schwab M. E. (2014). Finding an optimal rehabilitation paradigm after stroke: Enhancing fiber growth and training of the brain at the right moment. *Front. Hum. Neurosci.* 8:381. 10.3389/fnhum.2014.00381 25018717 PMC4072965

[B259] Wahl A. S. Buchler U. Brandli A. Brattoli B. Musall S. Kasper H. (2017). Optogenetically stimulating intact rat corticospinal tract post-stroke restores motor control through regionalized functional circuit formation. *Nat. Commun.* 8:1187. 10.1038/s41467-017-01090-6 29084962 PMC5662731

[B260] Wahl A. S. Omlor W. Rubio J. C. Chen J. L. Zheng H. Schroter A. (2014). Neuronal repair. Asynchronous therapy restores motor control by rewiring of the rat corticospinal tract after stroke. *Science* 344 1250–1255. 10.1126/science.1253050 24926013

[B261] Wang X. Liu Y. Li X. Zhang Z. Yang H. Zhang Y. (2017). Deconstruction of corticospinal circuits for goal-directed motor skills. *Cell* 171 440–455.e414. 10.1016/j.cell.2017.08.014 28942925 PMC5679421

[B262] Wang Z. Maunze B. Wang Y. Tsoulfas P. Blackmore M. G. (2018). Global connectivity and function of descending spinal input revealed by 3D microscopy and retrograde transduction. *J. Neurosci.* 38 10566–10581. 10.1523/JNEUROSCI.1196-18.2018 30341180 PMC6284107

[B263] Wang Z. Reynolds A. Kirry A. Nienhaus C. Blackmore M. G. (2015). Overexpression of Sox11 promotes corticospinal tract regeneration after spinal injury while interfering with functional recovery. *J. Neurosci.* 35 3139–3145. 10.1523/JNEUROSCI.2832-14.2015 25698749 PMC4331631

[B264] Wang Z. Romanski A. Mehra V. Wang Y. Brannigan M. Campbell B. C. (2022). Brain-wide analysis of the supraspinal connectome reveals anatomical correlates to functional recovery after spinal injury. *Elife* 11:e76254. 10.7554/eLife.76254 35838234 PMC9345604

[B265] Weidner N. Abel R. Maier D. Rohl K. Rohrich F. Baumberger M. (2025). Safety and efficacy of intrathecal antibodies to Nogo-A in patients with acute cervical spinal cord injury: A randomised, double-blind, multicentre, placebo-controlled, phase 2b trial. *Lancet Neurol.* 24 42–53. 10.1016/S1474-4422(24)00447-2 39706632

[B266] Weidner N. Ner A. Salimi N. Tuszynski M. H. (2001). Spontaneous corticospinal axonal plasticity and functional recovery after adult central nervous system injury. *Proc. Natl. Acad. Sci. U S A.* 98 3513–3518. 10.1073/pnas.051626798 11248109 PMC30684

[B267] Weishaupt N. Blesch A. Fouad K. (2012). BDNF: The career of a multifaceted neurotrophin in spinal cord injury. *Exp. Neurol.* 238 254–264. 10.1016/j.expneurol.2012.09.001 22982152

[B268] Welniarz Q. Dusart I. Roze E. (2017). The corticospinal tract: Evolution, development, and human disorders. *Dev. Neurobiol.* 77 810–829. 10.1002/dneu.22455 27706924

[B269] Wen T. C. Lall S. Pagnotta C. Markward J. Gupta D. Ratnadurai-Giridharan S. (2018). Plasticity in one hemisphere, control from two: Adaptation in descending motor pathways after unilateral corticospinal injury in neonatal rats. *Front. Neural Circuits* 12:28. 10.3389/fncir.2018.00028 29706871 PMC5906589

[B270] Whishaw I. Q. Gorny B. (1994). Arpeggio and fractionated digit movements used in prehension by rats. *Behav. Brain Res.* 60 15–24. 10.1016/0166-4328(94)90058-2 8185848

[B271] Whishaw I. Q. Gorny B. Sarna J. (1998). Paw and limb use in skilled and spontaneous reaching after pyramidal tract, red nucleus and combined lesions in the rat: Behavioral and anatomical dissociations. *Behav. Brain Res.* 93 167–183. 10.1016/s0166-4328(97)00152-6 9659998

[B272] Whishaw I. Q. Pellis S. M. Pellis V. C. (1992). A behavioral study of the contributions of cells and fibers of passage in the red nucleus of the rat to postural righting, skilled movements, and learning. *Behav. Brain Res.* 52 29–44. 10.1016/s0166-4328(05)80322-5 1472285

[B273] Whishaw I. Q. Tomie J. A. Ladowsky R. L. (1990). Red nucleus lesions do not affect limb preference or use, but exacerbate the effects of motor cortex lesions on grasping in the rat. *Behav. Brain Res.* 40 131–144. 10.1016/0166-4328(90)90005-y 2285474

[B274] Winter C. C. Jacobi A. Su J. Chung L. van Velthoven C. T. J. Yao Z. (2023). A transcriptomic taxonomy of mouse brain-wide spinal projecting neurons. *Nature* 624 403–414. 10.1038/s41586-023-06817-8 38092914 PMC10719099

[B275] Xie J. Feng R. Chen Y. Gao L. (2023). Morphological analysis of descending tracts in mouse spinal cord using tissue clearing, tissue expansion and tiling light sheet microscopy techniques. *Sci. Rep.* 13:16445. 10.1038/s41598-023-43610-z 37777565 PMC10542777

[B276] Yang L. Martin J. H. (2023). Effects of motor cortex neuromodulation on the specificity of corticospinal tract spinal axon outgrowth and targeting in rats. *Brain Stimul.* 16 759–771. 10.1016/j.brs.2023.04.014 37094762 PMC10501380

[B277] Yang Y. Chen X. Yang C. Liu M. Huang Q. Yang L. (2024). Chemogenetic stimulation of intact corticospinal tract during rehabilitative training promotes circuit rewiring and functional recovery after stroke. *Exp. Neurol.* 371:114603. 10.1016/j.expneurol.2023.114603 37923187

[B278] Yasui Y. Yokota S. Ono K. Tsumori T. (2001). Projections from the red nucleus to the parvicellular reticular formation and the cervical spinal cord in the rat, with special reference to innervation by branching axons. *Brain Res.* 923 187–192. 10.1016/s0006-8993(01)03196-1 11743987

[B279] Yiu G. He Z. (2006). Glial inhibition of CNS axon regeneration. *Nat. Rev. Neurosci.* 7 617–627. 10.1038/nrn1956 16858390 PMC2693386

[B280] Zagon A. Bacon S. J. (1991). Evidence of a monosynaptic pathway between cells of the ventromedial medulla and the motoneuron pool of the thoracic spinal cord in rat: Electron microscopic analysis of synaptic contacts. *Eur. J. Neurosci.* 3 55–65. 10.1111/j.1460-9568.1991.tb00811.x 12106269

[B281] Zai L. Ferrari C. Subbaiah S. Havton L. A. Coppola G. Strittmatter S. (2009). Inosine alters gene expression and axonal projections in neurons contralateral to a cortical infarct and improves skilled use of the impaired limb. *J. Neurosci.* 29 8187–8197. 10.1523/JNEUROSCI.0414-09.2009 19553458 PMC2856695

[B282] Zareen N. Yung H. Kaczetow W. Glattstein A. Mazalkova E. Alexander H. (2024). Molecular signaling predicts corticospinal axon growth state and muscle response plasticity induced by neuromodulation. *Proc. Natl. Acad. Sci. U S A.* 121:e2408508121. 10.1073/pnas.2408508121 39536089 PMC11588127

[B283] Z’Graggen W. J. Fouad K. Raineteau O. Metz G. A. Schwab M. E. Kartje G. L. (2000). Compensatory sprouting and impulse rerouting after unilateral pyramidal tract lesion in neonatal rats. *J. Neurosci.* 20 6561–6569. 10.1523/JNEUROSCI.20-17-06561.2000 10964961 PMC6772991

[B284] Z’Graggen W. J. Metz G. A. Kartje G. L. Thallmair M. Schwab M. E. (1998). Functional recovery and enhanced corticofugal plasticity after unilateral pyramidal tract lesion and blockade of myelin-associated neurite growth inhibitors in adult rats. *J. Neurosci.* 18 4744–4757. 10.1523/JNEUROSCI.18-12-04744.1998 9614248 PMC6792708

[B285] Zhang C. Zou Y. Li K. Li C. Jiang Y. Sun J. (2018). Different effects of running wheel exercise and skilled reaching training on corticofugal tract plasticity in hypertensive rats with cortical infarctions. *Behav. Brain Res.* 336 166–172. 10.1016/j.bbr.2017.09.002 28882693

[B286] Zhang Z. Su J. Tang J. Chung L. Page J. C. Winter C. C. (2024). Spinal projecting neurons in rostral ventromedial medulla co-regulate motor and sympathetic tone. *Cell* 187 3427–3444.e21. 10.1016/j.cell.2024.04.022 38733990 PMC11193620

[B287] Zholudeva L. V. Abraira V. E. Satkunendrarajah K. McDevitt T. C. Goulding M. D. Magnuson D. S. K. (2021). Spinal interneurons as gatekeepers to neuroplasticity after injury or disease. *J. Neurosci.* 41 845–854. 10.1523/JNEUROSCI.1654-20.2020 33472820 PMC7880285

[B288] Zingg B. Chou X. L. Zhang Z. G. Mesik L. Liang F. Tao H. W. (2017). AAV-mediated anterograde transsynaptic tagging: Mapping corticocollicular input-defined neural pathways for defense behaviors. *Neuron* 93 33–47. 10.1016/j.neuron.2016.11.045 27989459 PMC5538794

[B289] Zingg B. Peng B. Huang J. Tao H. W. Zhang L. I. (2020). Synaptic specificity and application of anterograde transsynaptic AAV for probing neural circuitry. *J. Neurosci.* 40 3250–3267. 10.1523/JNEUROSCI.2158-19.2020 32198185 PMC7159884

[B290] Zorner B. Bachmann L. C. Filli L. Kapitza S. Gullo M. Bolliger M. (2014). Chasing central nervous system plasticity: The brainstem’s contribution to locomotor recovery in rats with spinal cord injury. *Brain* 137 1716–1732. 10.1093/brain/awu078 24736305

